# Associations Between 24-Hour Movement Behaviors and Anthropometric and Body Composition Indicators in Ecuadorian Preschool Children: SUNRISE Pilot Study

**DOI:** 10.3390/children13091129

**Published:** 2026-08-24

**Authors:** Emily Cisneros-Vásquez, Estela Jiménez-López, Rodrigo Yáñez-Sepúlveda, Héctor Gutiérrez-Espinoza, Jorge Olivares-Arancibia, Pedro Antonio Sánchez-Miguel, Masoud Rahmati, Dong Keon Yon, Lee Smith, José Francisco López-Gil

**Affiliations:** 1 School of Medicine, Universidad Espíritu Santo, Samborondón 0901952, Ecuador; emily.sofy@hotmail.com; 2 Department of Health Research, Icen Cognis, 38370 La Matanza de Acentejo, Spain; 3 Health and Social Research Center, Universidad de Castilla-La Mancha, 13071 Cuenca, Spain; estela.jimenezlopez@uclm.es; 4 Department of Nursing, University of Castilla-La Mancha, 13071 Cuenca, Spain; 5 Faculty of Education and Social Sciences, Universidad Andres Bello, Viña del Mar 8370035, Chile; rodrigo.yanez.s@unab.cl; 6 Faculty of Education, Universidad Autónoma de Chile, Santiago 7500912, Chile; hector.gutierrez@uautonoma.cl; 7 AFySE Group, Research in Physical Activity and School Health, School of Physical Education, Faculty of Education, Universidad de las Américas, Santiago 7500975, Chile; jolivares@udla.cl; 8 Departamento de Didáctica de la Expresión Musical, Plástica y Corporal, Grupo de Investigación Análisis Didáctico y Comportamental del Deporte (ADICODE), Facultad de Formación del Profesorado, Universidad de Extremadura, 10003 Cáceres, Spain; pesanchezm@unex.es; 9 CEReSS-Health Service Research and Quality of Life Center, Aix-Marseille University, 13284 Marseille, France; masoud.rahmati@univ-amu.fr; 10 Clinical Research Unit, Délégation à la Recherche Clinique et a l’Innovation du GHT, Center Hospitalier Intercommunal de Toulon La Seyne-sur-Mer, 83100 Toulon, France; 11 Center for Digital Health, Medical Science Research Institute, College of Medicine, Kyung Hee University, Seoul 02447, Republic of Korea; yonkkang@gmail.com; 12 Department of Precision Medicine, College of Medicine, Kyung Hee University, Seoul 02447, Republic of Korea; 13 Department of Pediatrics, Kyung Hee University Medical Center, College of Medicine, Kyung Hee University, Seoul 02447, Republic of Korea; 14 Centre for Health Performance and Wellbeing, Anglia Ruskin University, Cambridge CB1 1PT, UK; lee.smith@aru.ac.uk; 15 Department of Public Health, Faculty of Medicine, Biruni University, Istanbul 34015, Turkey; 16 Faculty of Health Sciences, Universidad Tecnológica Atlántico Mediterráneo—UTAMED, 29590 Malaga, Spain

**Keywords:** childhood, obesity, daily movement behaviors, physical activity, sleep, screen time, Ecuador

## Abstract

**Highlights:**

**What are the main findings?**

- Exploratory unadjusted analyses identified associations between shorter accelerometer-measured sleep duration and less favorable anthropometric indicators in Ecuadorian preschool children, but these associations attenuated after covariate adjustment.
- Moderate-to-vigorous physical activity and total physical activity were positively associated with growth indicators and fat-free mass in unadjusted analyses; these associations were attenuated in complementary adjusted models.
- Accelerometer-derived movement behaviors showed more consistent associations with anthropometric and body composition indicators than parent-reported measures.

**What are the implications of the main findings?**

- Preliminary sleep/moderate-to-vigorous physical activity–anthropometry associations attenuated after adjustment and should be interpreted as hypothesis-generating.
- Objective assessment of 24-hour movement behaviors may provide more robust evidence than parent-reported measures in early childhood research.
- Larger longitudinal studies are needed to confirm these exploratory findings and support evidence-based interventions in Ecuador and other low- and middle-income settings.

**Abstract:**

Objective: To analyze associations between 24-hour movement behaviors (and guideline adherence) and anthropometric and body composition indicators in Ecuadorian preschoolers using data from the International Study of Movement Behaviors in the Early Years (SUNRISE) pilot study. Methods: This secondary analysis utilized data from the primary study conducted in 108 children aged 3–4 years (57.4% girls; 50.9% urban). Anthropometry (weight, height, and body mass index [BMI]) was assessed using standardized protocols during the pilot study, whereas body composition indicators (fat-free mass [FFM], fat mass [FM], and body fat percentage) were estimated using a validated anthropometric prediction equation. Movement behaviors were assessed via triaxial accelerometry (ActiGraph GT3X+ or GT3X-BT, Pensacola, FL, USA) over seven days, complemented by parent-reported questionnaires. Exploratory unadjusted correlation analyses were performed using Spearman’s rho (ρ) for continuous/ordinal variables, and Pearson’s correlations (r), mathematically equivalent to point-biserial correlations (r_pb_), for dichotomous–continuous associations. Results: In exploratory unadjusted analyses, device-measured longer sleep duration showed small-to-moderate inverse associations with weight (ρ = −0.33, *p* = 0.001), BMI (ρ = −0.28, *p* = 0.006), and FFM (ρ = −0.37, *p* < 0.001). Higher moderate-to-vigorous physical activity showed moderate positive associations with height (ρ = 0.32, *p* = 0.002), weight (ρ = 0.40, *p* < 0.001), and FFM (ρ = 0.44, *p* < 0.001). In complementary multivariable models, the associations between device-measured sleep or moderate-to-vigorous physical activity (MVPA) and the pre-specified anthropometric and body composition outcomes were attenuated after adjustment for age, sex, area of residence, and, where applicable, height, and were generally not statistically significant. Conclusions: In this exploratory secondary analysis of the SUNRISE Pilot Study Ecuador, unadjusted analyses identified associations between specific 24-hour movement behaviors, particularly sleep duration and MVPA, and anthropometric and body composition indicators. However, these associations were attenuated after covariate adjustment. The findings should therefore be interpreted as hypothesis-generating and require confirmation in larger longitudinal studies.

## 1. Introduction

The prevalence of overweight and obesity among children has shown a sustained global increase over recent decades, becoming a priority public health concern due to its substantial burden of morbidity, mortality, and associated economic costs [1,2,3]. Global estimates indicate a sharp rise in childhood overweight and obesity, with 35 million children under five affected worldwide in 2024, most notably in low- and middle-income countries [4]. In South America, childhood overweight prevalence reached 9.7% in 2022 [5]. In Ecuador, national estimates and synthesis studies report a high burden across age groups, with obesity rates ranging from 8.1% in preschoolers to nearly 13% in older youth [6,7]. This trend highlights the urgent need for early-life preventive strategies in resource-limited contexts [8].

The clinical implications of childhood obesity are extensive and multifactorial [9,10,11]. It has been associated with a higher probability of developing metabolic and cardiovascular comorbidities, including insulin resistance, type 2 diabetes, dyslipidemia, hypertension, and metabolic syndrome [4,12,13,14,15,16]. Alterations in endocrine and pubertal development, such as precocious puberty and obstructive sleep apnea, have also been reported, along with psychosocial conditions including depression, anxiety, low self-esteem, body image distortion, and impaired social interaction [15,17,18]. Moreover, childhood obesity is linked to an approximately fivefold greater likelihood of persisting into adulthood [14], with implications for chronic disease, premature mortality, and substantially increased direct and indirect healthcare costs [3,19].

The integrated 24-hour movement behavior model has emerged as a robust conceptual framework for promoting child health and preventing obesity [20,21]. This approach considers three interdependent components (i.e., physical activity, sedentary behaviors (particularly screen time), and sleep) [20,21]. Within this framework, the World Health Organization (WHO) 24-hour movement behavior guidelines for children under 5 years of age recommend at least 180 min of daily physical activity, of which a minimum of 60 min should be of moderate- to vigorous-intensity; limiting recreational screen time to less than 60 min per day; and ensuring 10–13 h of good-quality sleep [22]. Although simultaneous adherence to these guidelines has been associated with benefits in body composition, cardiometabolic markers, mental health, cognitive development, and quality of life [20,23,24,25,26], international evidence indicates low adherence rates: 11.3% (n = 11,768; 23 countries) and 14.3% (n = 7017; 33 countries), with the lowest values in South America [27,28].

Despite the growing body of evidence on the relevance of meeting the 24-hour movement guidelines, most available studies have been conducted in high-income countries [20,27], limiting the generalizability of their findings to other socioeconomic contexts. Although recent evidence from the International Study of Movement Behaviors in the Early Years (SUNRISE) has expanded the characterization of 24-hour movement behaviors among preschool children in Latin America [27,28,29,30], country-specific evidence remains limited [31], particularly regarding the associations between these behaviors and anthropometric and body composition indicators. To our knowledge, to date, no such analyses have been conducted among Ecuadorian preschoolers, highlighting a critical gap in the development of evidence-based interventions and policies. This is particularly relevant given that early childhood is a period of high biological and behavioral changes, during which lifestyle habits can be established with long-term health consequences [32].

Furthermore, although body mass index (BMI) is widely used as a proxy for child growth, it lacks the specificity required to distinguish between fat mass and fat-free mass, limiting its capacity to capture subtle variations in body composition during periods of rapid growth [33]. The preschool years represent a critical physiological window characterized by dynamic changes in tissue accretion, musculoskeletal development, and linear growth [34,35]. Therefore, examining anthropometric measures alongside estimated body composition indicators, such as fat mass, fat-free mass, and body fat percentage, may provide a more comprehensive and biologically relevant framework to understand how daily 24-hour movement behaviors influence early cardiometabolic health and developmental trajectories beyond simple gross weight or BMI metrics [21,36].

Against this backdrop, the SUNRISE project has been established as a global surveillance system for these behaviors, with particular emphasis on low- and middle-income countries [37]. In 2024, the SUNRISE pilot study was implemented in Ecuador to evaluate the feasibility and acceptability of the international protocol within the national context. As part of this initiative, preliminary descriptive findings revealed that only 11.8% of the preschoolers met the recommendations for sleep, physical activity, and screen time, whereas 16.1% met none [38]. Given the high burden of childhood obesity, the low adherence to the guidelines, and the absence of prior studies in the country, the present study aimed to determine associations between adherence to the three 24-hour movement guidelines and anthropometric and body composition indicators in Ecuadorian children aged 3–4 years, using data from the SUNRISE Pilot Study Ecuador. Based on previous evidence, we hypothesized that longer sleep duration, higher levels of physical activity, lower recreational screen time, and greater adherence to the WHO 24-hour movement guidelines would be associated with more favorable anthropometric and body composition profiles, particularly greater fat-free mass and lower adiposity.

## 2. Materials and Methods

### 2.1. Study Design and Population

#### 2.1.1. Study Type

The present study performs a secondary analysis of data obtained from the SUNRISE Pilot Study Ecuador, which employed a cross-sectional observational design and recruited 108 Ecuadorian children aged 3 to 4 years. According to the SUNRISE protocol, a minimum of 100 participants were required to evaluate the feasibility and acceptability of the study procedures as a preliminary step before implementing the main national study. The sample was therefore deemed sufficient for this purpose. Sex balance and an equitable distribution between urban and rural settings (approximately 50 children per group) were ensured during the original recruitment. All primary methodological procedures were conducted in accordance with the standardized guidelines described by Okely et al. [37], and the reporting of this cross-sectional study followed the Strengthening the Reporting of Observational Studies in Epidemiology (STROBE) statement (Supplementary Material S1).

#### 2.1.2. Study Population and Sampling Design

Data collection for the pilot study was conducted between September and December 2024. A nonprobabilistic cluster sampling design based on convenience was employed. Public and private early childhood education centers located in Quito, and its surroundings (Pichincha, Ecuador) were defined as the primary sampling units. The target population comprised children aged 3 to 4 years enrolled in these centers whose parents or legal guardians provided written informed consent during that original phase. Exclusion criteria applied in the fieldwork comprised medical conditions or disabilities that could interfere with wearable device data collection, compromise motor or executive function assessments, or pose safety risks. To ensure sample variability, a maximum of 20 children per center was established, due to low eligibility in certain institutions, additional centers were progressively included, totaling 11 educational centers involved in the pilot project.

Institutional approvals and recruitment sessions were carried out in the baseline fieldwork by an interdisciplinary team of eight researchers, specialized in Education, Sport Sciences, Pediatrics, Public Health, Epidemiology, and Nutrition, trained by the SUNRISE Coordinating Centre. All study materials underwent translation and cross-cultural adaptation prior to fieldwork.

#### 2.1.3. Ethical Approval

The general study protocol (ID 2018/044; ID 2019/378, with approval granted on 17 December 2019) received ethical approval from the Human Research Ethics Committee (HREC) of the University of Wollongong (UOW), Australia. Additional ethical clearance for the SUNRISE Pilot Study Ecuador was granted by the *Comité de Ética de Investigación en Seres Humanos de la Universidad Espíritu Santo* (ID C-UEES-25-13, update on 16 July 2025). All procedures involving human participants, performed during that primary data collection phase, were in accordance with the ethical standards of the institutional and national research committees and with the Helsinki Declaration and its later amendments. The present secondary analysis utilized the de-identified dataset generated from this approved pilot study.

### 2.2. Measurements

All variables analyzed in this study were derived from the dataset collected during the SUNRISE Ecuador pilot fieldwork following standardized protocols [37] and entered into the Research Electronic Data Capture (REDCap) system (Vanderbilt University, Nashville, TN, USA) [39] for subsequent secondary analysis.

#### 2.2.1. Anthropometry

Anthropometric measurements of stature and body weight were measured, during the pilot study, using a high-precision portable stadiometer (SECA 213, GmbH & Co. KG, Hamburg, Germany) and precalibrated electronic scales (SECA 876, GmbH & Co. KG, Hamburg, Germany), following the standardized procedures outlined in the WHO guidelines [40]. Each measurement was recorded twice, and a third reading was taken if the discrepancy exceeded 0.5 cm for height or 0.25 kg for weight to ensure accuracy and reliability. Intra-observer technical error of measurement (TEM) and a two-way random-effects, absolute-agreement, single-measure intraclass correlation coefficient ICC_(2.1)_ were computed from the first and second readings in all 108 children. For this secondary analysis, BMI z-scores were computed via the WHO Anthro software (version 1.0.4; WHO, Geneva, Switzerland) to classify nutritional status according to the WHO International Growth Reference Standards [41].

Fat-free mass (FFM; kg), fat mass (FM; kg) and body fat percentage were estimated from the dataset’s weight, height and demographic characteristics (age, sex and ethnicity) using the predictive equation developed and validated by Hudda et al. [42]. Body fat percentage was calculated as (fat mass/body weight) × 100. These equation-based estimates are not equivalent to criterion methods such as dual-energy X-ray absorptiometry and should be interpreted as derived anthropometric indicators of body composition.

#### 2.2.2. Twenty-Four-Hour Movement Behaviors

Twenty-four-hour movement behaviors, including physical activity, sedentary time, and sleep, were objectively assessed in the pilot study via triaxial accelerometry [34]. Each participating child was provided with an ActiGraph GT3X+ or GT3X-BT device (ActiGraph LLC, Pensacola, FL, USA), configured to initiate data acquisition at 00:00 hours on the day of distribution. The devices were secured at the waist and set to sample at a frequency of 30 Hz. The participants were instructed to wear the accelerometers continuously over a seven-day monitoring period, removing them only during water-based activities such as bathing or swimming. Adherence to the wear protocol was supervised by school personnel during academic hours and by parents or guardians during out-of-school time.

The raw accelerometer data were extracted and processed via ActiLife software (version 6.12.1). The data were aggregated into 15 s epochs and processed via a low-frequency extension filter to enhance the detection of lower-intensity activities. A recording was deemed valid if it contained at least one full day of monitoring with a minimum of six hours of wear time during waking hours, as verified through manual data inspection [43]. This validity criterion was predefined by the standardized SUNRISE protocol to optimize feasibility and participant retention in preschool populations, despite the potential reduction in the reliability of habitual movement behavior estimates relative to more stringent wear-time criteria [37].

To isolate daytime behaviors, a standardized time window from 08:00 to 22:20 was applied on the basis of typical sleep and wake patterns reported in the target population. Nonwear periods were defined as sequences of at least 20 consecutive minutes registering zero counts and were excluded from further analysis [44]. Physical activity intensities were classified according to validated cutoff points: sedentary (<800 counts per minute [CPM]), low intensity (LPA; 800–1679 CPM), moderate intensity (MPA; 1680–3367 CPM), vigorous intensity (VPA; ≥3368 CPM), and moderate-to-vigorous physical activity (MVPA; ≥1680 CPM) [45,46]. Device-measured total sleep duration reflects all sleep epochs detected over the 24-hour monitoring period (including daytime naps during valid wear), processed in ActiLife following the SUNRISE protocol. Daytime physical activity intensities were estimated within the 08:00–22:20 window; sleep and sedentary time outside this window were classified separately to derive total daily sleep duration.

Complementary to device-based assessments, parent-reported movement behaviors were collected via the standardized SUNRISE parent questionnaire (see Parent Questionnaire subsection).

#### 2.2.3. Parent Questionnaire

The primary caregiver completed a self-administered questionnaire during the baseline fieldwork to capture habitual movement-related behaviors. The questionnaire used to assess movement behaviors is part of the standardized SUNRISE parent questionnaire developed and published by Okely et al. [37]. An English-language version of the adapted questionnaire used in the Ecuadorian pilot is provided in Supplementary Material S2. The instrument, which required approximately 15–20 min to complete, collected detailed information on the child’s physical activity levels, sleep-wake patterns (including nap duration), screen exposure, and other sedentary practices [37]. Sleep duration was estimated on the basis of caregiver responses to the following question: “How many hours did your child usually sleep in a 24-hour day (including naps)?” Screen time was estimated via the following question: “Over the past week, how many hours per 24-hour day did your child typically spend using electronic devices such as smartphones, tablets, video games, or watching TV or online videos while sitting or lying down?” Caregivers were instructed to provide precise estimates, rounded to the nearest minute, to facilitate accurate characterization of sleep and sedentary behaviors and to complement accelerometry-derived measures. This device-use item was analyzed as parent-reported sitting time (minutes per day) and dichotomized as the WHO screen-time guideline (ST guideline; ≤60 min/day). Restrained sitting was assessed as a parent-reported yes/no item (whether the child was kept immobile for more than one hour, for example in a car seat or similar) and was analyzed as the restrained-sitting guideline, not as a continuous duration.

In addition to the assessment of children’s 24-hour movement behaviors, standardized and validated instruments were administered to parents or primary caregivers to identify factors associated with such behaviors within a socioecological framework. This approach facilitated the examination of dynamic interactions between the child and both social and physical environmental contexts [47]. Sociodemographic variables were captured via a modified version of the WHO STEPwise approach to noncommunicable Diseases (NCD) risk factor surveillance (STEPS) instrument [48].

#### 2.2.4. Statistical Analysis

**Figure 1:**
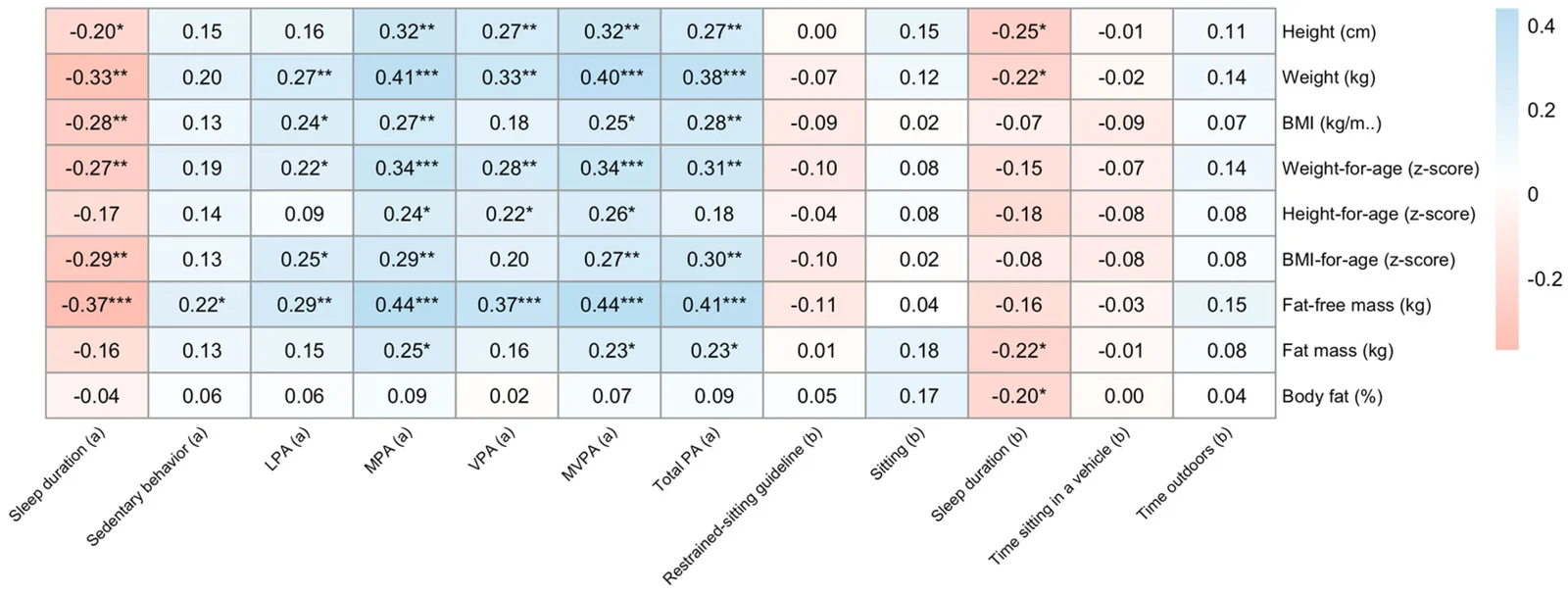
Associations between 24-hour movement behaviors and body composition among Ecuadorian preschoolers. Colors: blue indicates a positive association and red a negative association. Color intensity reflects the magnitude of the correlation. Asterisks denote statistical significance based on unadjusted *p*-values: * *p* < 0.05, ** *p* < 0.01, *** *p* < 0.001; cells without asterisks were not significant. Abbreviations: BMI, body mass index; PA, physical activity. Measurement sources: (a) Measured with an accelerometer, (b) Measured with a parent questionnaire. In column (b), restrained sitting denotes the restrained-sitting guideline (yes/no), not a continuous duration in minutes. *p*-values are uncorrected for multiple comparisons.

For this secondary analysis, statistical analyses were conducted via R software (version 4.4.1; R Foundation for Statistical Computing, Vienna, Austria) and RStudio (version 2024.09.0+375; Posit, Boston, MA, USA). The normality of continuous variables was assessed through graphical inspection (Q–Q plots and density plots) and the Shapiro–Wilk test. The homogeneity of variance was evaluated via Levene’s test. For continuous variables, data were reported as the median and interquartile range (IQR), with mean ± standard deviation also provided in Table 1 to facilitate comparability with previous studies. For categorical variables, data were presented as absolute frequencies and relative proportions. As the primary exploratory analysis, Spearman’s rho (ρ) was applied for correlations involving continuous or ordinal variables given the frequent non-normal distribution of the data. Pearson’s correlation coefficient (r) was used when examining associations between dichotomous and continuous variables (e.g., excess weight, obesity, or guideline adherence), which are mathematically equivalent to point-biserial correlations (r_pb_). Primary correlation analyses were performed at the unadjusted level. Results are presented in heatmaps for: (i) 24-hour movement behaviors and body composition indicators, (ii) 24-hour movement behaviors and weight status, and (iii) adherence to 24-hour movement guidelines and body composition indicators. Missing data were handled using complete-case pairwise deletion for each correlation (Supplementary Table S3). No imputation was applied. Because numerous exploratory correlations were tested without pre-specified primary hypotheses, *p*-values are uncorrected for multiple comparisons. Approximately 108 pairwise correlations were examined in Figure 1 alone; under a global null at α = 0.05, about 5.4 associations would be expected to reach statistical significance by chance. Accordingly, nominally significant findings should be interpreted cautiously and as hypothesis-generating. As a complementary revision analysis, we pre-specified a limited set of primary associations (device-measured sleep duration and MVPA with BMI-for-age z-score, weight, fat-free mass and fat mass) and fitted multivariable linear models adjusted for age, sex and area of residence; models of weight and body composition additionally adjusted for height. Cluster-robust standard errors were computed by educational center (n = 11) to account for recruitment clustering (Supplementary Table S4). A sensitivity analysis restricted to children with ≥2 valid accelerometer days is presented in Supplementary Table S5. Descriptive sensitivity analyses for selected associations by sex and area of residence are reported in Supplementary Table S6. Statistical significance was set at two-sided *p* < 0.05. For each Spearman coefficient we now also report a 95% confidence interval obtained by Fisher’s z-transformation. In addition, the complete unfiltered pairwise matrix corresponding to Figure 1 (12 movement × 9 composition indicators; 108 tests) is provided in Supplementary Table S7, including n, ρ, 95% CI, unadjusted *p*-values and Benjamini–Hochberg q-values applied to the full family of tests (not to a pre-selected significant subset). Associations with |ρ| < 0.29 at n = 93 are interpreted as statistically fragile even when nominally significant, because the study is underpowered to detect effects of that magnitude reliably. Heatmaps remain the primary exploratory display. Table S7 supplies the interval and false discovery rate (FDR) information.

## 3. Results

A total of 108 participants were evaluated in this secondary analysis, with a median age of 4.0 years (IQR: 3.5–4.3). They were predominantly girls (57.4%), and the distribution across urban (50.9%) and rural (49.1%) settings was nearly equal. The z-scores for height-for-age and BMI-for-age had medians of −0.8 SD (standard deviation) (IQR: −1.5 to −0.1) and 0.7 SD (IQR: 0.2–1.3), respectively. The overall prevalence of excess weight was 38.9% (95% CI, confidence interval: 29.8–48.8), with overweight (31.5%) occurring more frequently than obesity (7.4%). The body composition parameters included a median FFM of 13.5 kg (IQR: 12.7–14.6), FM of 2.6 kg (IQR: 1.9–3.1), and body fat percentage of 15.7% (IQR: 12.5–18.4). Duplicate anthropometric readings in this sample showed high intra-observer reliability. A third height reading was protocol-indicated (|trial 1 − trial 2| > 0.5 cm) in 12/108 children (11.1%), and a third weight reading (|trial 1 − trial 2| > 0.25 kg) in 3/108 children (2.8%). TEM from the paired first and second readings was 0.26 cm for height (relative TEM 0.26%) and 0.075 kg for weight (relative TEM 0.46%). The corresponding ICC_(2,1)_ was 0.997 (95% CI 0.996–0.998) for height and 0.999 (95% CI 0.999–0.999) for weight. A complete characterization of the study population, covering sociodemographic, anthropometric, and body composition distributions and adherence to the 24-hour movement guidelines, is provided in Table 1.

Spearman’s correlations (ρ) revealed overall weak-to-moderate associations between 24-hour movement behaviors and anthropometric and body composition outcomes (Figure 1; continuous-to-continuous variables). Device-measured sleep duration showed an inverse association with height (ρ = −0.20, 95% CI −0.39 to −0.001, *p* = 0.049; Benjamini–Hochberg *q* = 0.120, not retained after FDR control) and weak-to-moderate inverse associations with weight (ρ = −0.33, 95% CI −0.50 to −0.14, *p* = 0.001, *q* = 0.012), BMI (ρ = −0.28, 95% CI −0.46 to −0.08, *p* = 0.006, *q* = 0.032), weight-for-age z-score (ρ = −0.27, 95% CI −0.45 to −0.07, *p* = 0.008, *q* = 0.035), BMI-for-age z-score (ρ = −0.29, 95% CI −0.46 to −0.09, *p* = 0.005, *q* = 0.031), and FFM (ρ = −0.37, 95% CI −0.53 to −0.18, *p* < 0.001, *q* = 0.004), with no significant associations for height-for-age, FM, or body fat percentage. Device-measured sedentary time was weakly correlated with FFM (ρ = 0.22, 95% CI 0.02 to 0.41, *p* = 0.032, *q* = 0.085; not retained after FDR control), whereas LPA showed weak positive associations with weight, BMI, weight-for-age, BMI-for-age, and FFM (ρ range = 0.22–0.29, all *p* < 0.05; associations with BMI, weight-for-age, and BMI-for-age were not retained after FDR control). VPA showed weak-to-moderate positive correlations with height (ρ = 0.27, *p* = 0.009, *q* = 0.035), weight (ρ = 0.33, *p* = 0.001, *q* = 0.012), weight-for-age (ρ = 0.28, *p* = 0.007, *q* = 0.035), height-for-age (ρ = 0.22, *p* = 0.037, *q* = 0.095; not retained after FDR control), and FFM (ρ = 0.37, *p* < 0.001, *q* = 0.004), whereas associations with BMI, BMI-for-age, FM, and body fat percentage were not significant. MPA, MVPA, and total physical activity (TPA) showed the largest correlations with weight (MPA: ρ = 0.41; MVPA: ρ = 0.40; TPA: ρ = 0.38; all *p* < 0.001) and FFM (MPA: ρ = 0.44; MVPA: ρ = 0.44, 95% CI 0.26 to 0.59; TPA: ρ = 0.41, 95% CI 0.22 to 0.56; all *p* < 0.001 and *q* ≤ 0.001). These variables were also associated with height, BMI, weight-for-age, and BMI-for-age (the MVPA–BMI association was not retained after FDR control), but not with body fat percentage. Associations with FM were smaller and were not retained after FDR control (largest: MPA, ρ = 0.25, *p* = 0.018, *q* = 0.058). Parent-reported behaviors yielded fewer and generally weak associations: reported sleep duration was inversely related to height (ρ = −0.25, *p* = 0.010, *q* = 0.038) and showed nominal inverse associations with weight (ρ = −0.22, *p* = 0.022, *q* = 0.066) and FM (ρ = −0.22, *p* = 0.024, *q* = 0.070) and body fat percentage (ρ = −0.20, *p* = 0.044, *q* = 0.110) that were not retained after FDR control, whereas parent-reported sitting time was not significantly associated with any body composition outcome. The complete unfiltered matrix with 95% CIs and Benjamini–Hochberg q-values is reported in Supplementary Table S7. Time spent sitting in a vehicle and time spent outdoors (n = 107) were not significantly associated with any body composition outcome.

**Figure 2:**
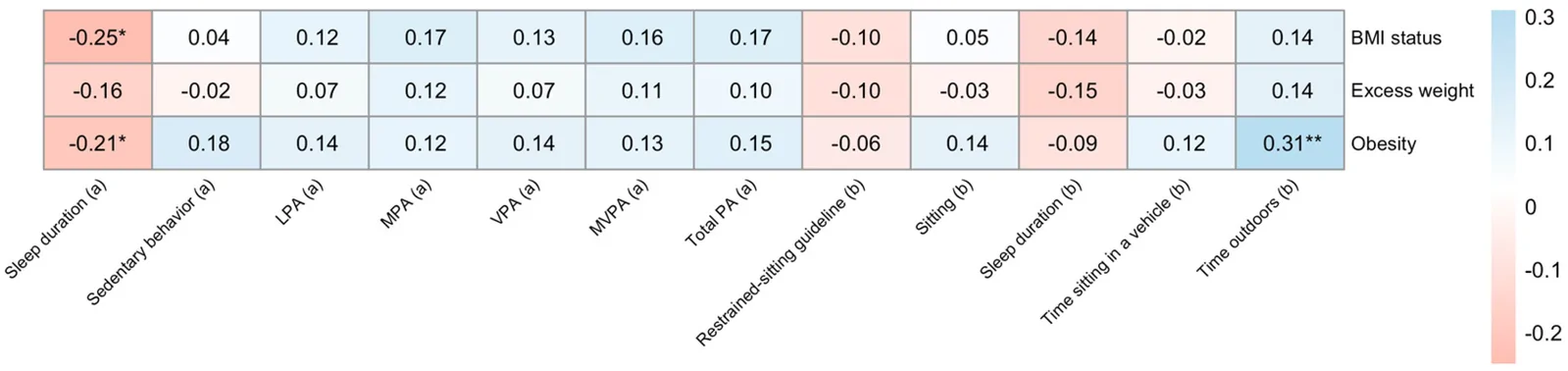
Associations between 24-hour movement behaviors and weight status among Ecuadorian preschoolers. Colors: blue indicates a positive association and red a negative association. Color intensity reflects the magnitude of the correlation. Asterisks denote statistical significance based on unadjusted *p*-values: * *p* < 0.05, ** *p* < 0.01; cells without asterisks were not significant. Abbreviations: BMI, body mass index; PA, physical activity. Measurement sources: (a) Measured with an accelerometer, (b) Measured with a parent questionnaire. In column (b), restrained sitting denotes the restrained-sitting guideline (yes/no), not a continuous duration in minutes. *p*-values are uncorrected for multiple comparisons.

Figure 2 presents associations between movement behaviors (continuous variables, together with the dichotomous restrained-sitting guideline) and weight-status categories, using Spearman’s ρ for ordinal BMI status and point-biserial correlations (r_pb_) for dichotomous excess weight and obesity. Device-measured sleep duration was inversely associated with BMI status (ρ = −0.25, *p* < 0.05) and obesity (r_pb_ = −0.21, *p* < 0.05), whereas the association with excess weight was not significant (r_pb_ = −0.16). No device-measured physical activity (LPA, MPA, VPA, MVPA, or TPA) or sedentary time variables were significantly associated with BMI status, excess weight, or obesity, with correlation coefficients ranging from −0.02 to 0.18. Parent-reported behaviors also demonstrated limited associations: reported sleep duration was negatively but non-significantly correlated with weight indicators (ρ range = −0.09 to −0.15), and the restrained-sitting guideline, parent-reported sitting time, and time spent sitting in a vehicle had coefficients close to zero. Time spent outdoors showed a positive association with obesity (r_pb_ = 0.31, *p* < 0.01). The correlation was based on n = 107 parent reports, of whom 8 had obesity. Time spent outdoors was not associated with body composition outcomes in Supplementary Table S7 (n = 107).

**Figure 3:**
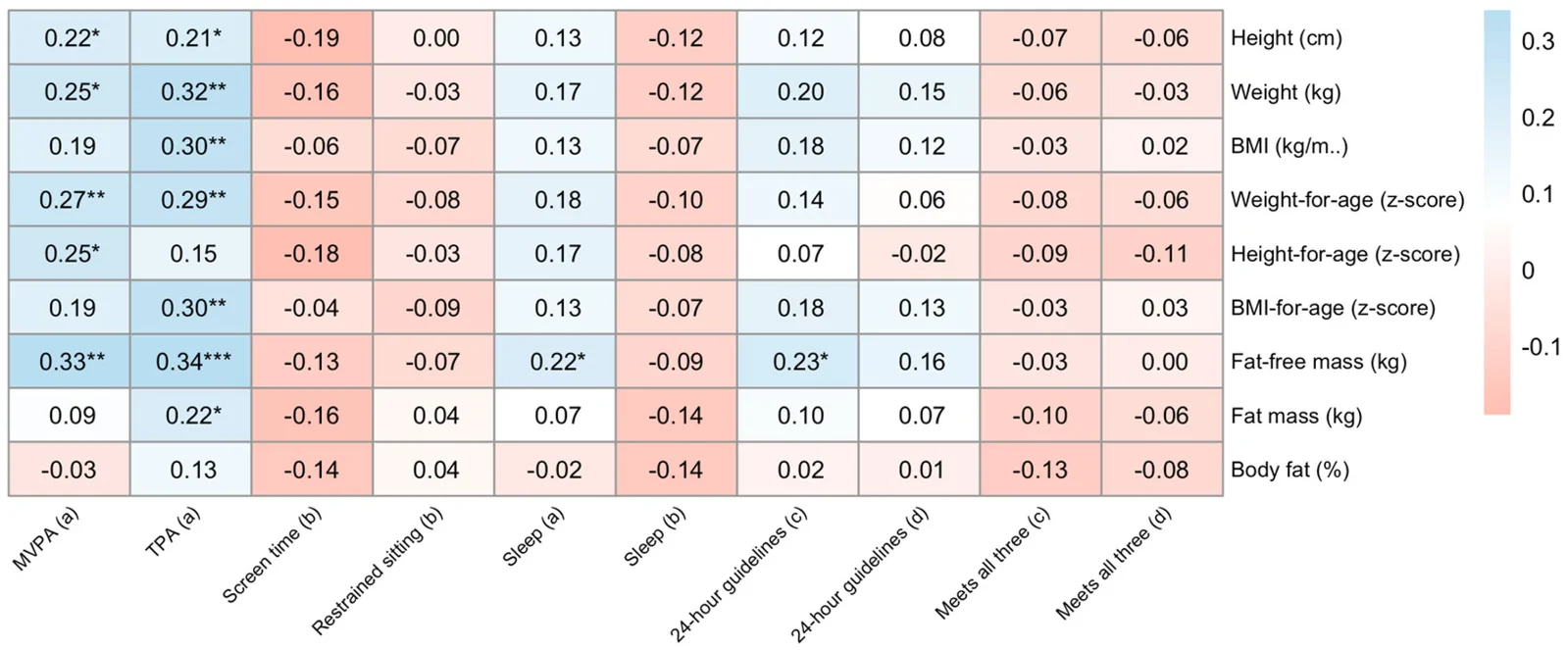
Compliance with the 24-hour movement guidelines and their relation to body composition among Ecuadorian preschoolers. Colors: blue denotes a positive association and red a negative association. Color intensity reflects the magnitude of the correlation. Asterisks indicate statistical significance based on unadjusted *p*-values: * *p* < 0.05, ** *p* < 0.01, *** *p* < 0.001; cells without asterisks were not significant. Abbreviations: BMI, body mass index; PA, physical activity. Measurement sources: (a) Measured with an accelerometer, (b) Measured with a parent questionnaire, (c) Moderate-to-vigorous physical activity, total physical activity and sleep guidelines measured with an accelerometer; screen time and restrained sitting guidelines measured with a parent questionnaire, (d) Moderate-to-vigorous physical activity and total physical activity guidelines measured with an accelerometer; screen time, restrained sitting and sleep guidelines measured with a parent questionnaire, According to the World Health Organization criteria. *p*-values are uncorrected for multiple comparisons.

Finally, as shown in Figure 3, adherence to 24-hour movement guidelines (dichotomous yes/no) was evaluated using point-biserial correlations (r_pb_) against body composition indicators. These coefficients are not interchangeable with the continuous correlations in Figure 1. Meeting the device-measured MVPA guideline was positively associated with height (r_pb_ = 0.22, *p* < 0.05), weight (r_pb_ = 0.25, *p* < 0.05), weight-for-age (r_pb_ = 0.27, *p* < 0.01), height-for-age (r_pb_ = 0.25, *p* < 0.05), and FFM (r_pb_ = 0.33, *p* < 0.01). Meeting the device-measured TPA guideline showed a similar pattern, including associations with height (r_pb_ = 0.21, *p* < 0.05), weight (r_pb_ = 0.32, *p* < 0.01), BMI (r_pb_ = 0.30, *p* < 0.01), weight-for-age (r_pb_ = 0.29, *p* < 0.01), BMI-for-age (r_pb_ = 0.30, *p* < 0.01), FFM (r_pb_ = 0.34, *p* < 0.001), and FM (r_pb_ = 0.22, *p* < 0.05). Meeting the parent-reported screen-time guideline (≤60 min/day) was not significantly associated with height (r_pb_ = −0.19, *p* = 0.051, n = 107), weight (r_pb_ = −0.16, *p* = 0.101), or height-for-age (r_pb_ = −0.18, *p* = 0.059). Continuous parent-reported sitting time was likewise not associated with height (ρ = 0.15, *p* = 0.124) or weight (ρ = 0.12, *p* = 0.234) (Figure 1; Supplementary Table S7). Additional findings included a positive association between meeting the device-measured sleep guideline and FFM (r_pb_ = 0.22, *p* < 0.05) and between accelerometer-based 24-hour guideline adherence and FFM (r_pb_ = 0.23, *p* < 0.05). No significant associations were observed for the restrained-sitting guideline, the parent-reported sleep guideline, or parent-reported operationalizations of guideline adherence.

In complementary multivariable models (Supplementary Table S4), associations between device-measured sleep or MVPA and the pre-specified anthropometric/body composition outcomes attenuated after adjustment for age, sex, area of residence and, where applicable, height, and were generally not statistically significant at conventional thresholds (MVPA and BMI-for-age z-score: *p* = 0.050), although the direction of estimates was largely preserved. Findings were similar when analyses were restricted to children with ≥2 valid accelerometer days (Supplementary Table S5). 

## 4. Discussion

This study provides preliminary evidence on the associations between 24-hour movement guidelines and anthropometric and body composition indicators in Ecuadorian preschoolers using data from the SUNRISE Pilot Study Ecuador, extending the evidence available from Latin America and contributing to the growing literature from low- and middle-income countries. In exploratory unadjusted analyses, shorter accelerometer-measured sleep duration was associated with higher BMI-related indicators and greater FFM, while moderate-to-vigorous and total physical activity were positively associated with growth parameters and FFM. These associations were attenuated in complementary multivariable models after adjustment for relevant covariates. Neither meeting the parent-reported screen-time guideline nor continuous parent-reported sitting time was significantly associated with height or weight. Other parental reports showed limited and inconsistent associations.

These patterns align with international evidence reporting inverse associations between sleep duration and adiposity [24,49,50] and between MVPA and lean mass development [51,52,53,54,55]. Excessive screen exposure has been linked to adverse growth outcomes in other pediatric samples [56,57]. The absence of significant associations between device-measured sedentary time and adiposity is also in line with systematic reviews reporting heterogeneous results [58]. In this sample, parent-reported sitting time and meeting the screen-time guideline were likewise not associated with anthropometric outcomes. Unadjusted analyses did show inverse associations of device-measured sleep duration with BMI and obesity, and an unexpected positive association of parent-reported time outdoors with obesity, alongside further correlations with body composition, particularly FFM. These patterns underscore the added value of assessing composition alongside conventional weight-status indicators. The observed associations between accelerometer-based adherence and FFM provide novel insights from the Ecuadorian context, highlighting the added value of objective measurement methods. Nevertheless, results must be interpreted with caution given the study’s cross-sectional design, small sample size and non-probabilistic recruitment of the original pilot fieldwork. Complementary multivariable models adjusting for age, sex, area of residence and, where applicable, height showed attenuation of the main bivariate associations (Supplementary Table S4), suggesting that demographic factors and body size may partly account for the unadjusted correlations and limiting claims of independent behavioral effects.

The inverse associations between device-measured sleep duration and both adiposity and FFM add to the growing body of evidence that sleep has been consistently associated with weight regulation and body composition during early childhood [24,25,59]. Inadequate sleep has been linked to metabolic and hormonal dysregulation, including altered leptin and ghrelin concentrations, reduced insulin sensitivity, and elevated cortisol, all of which may be related to increased appetite, energy intake, and fat deposition [60,61,62,63]. Moreover, shorter sleep may be associated with differences in growth hormone secretion, which occurs predominantly during deep sleep and is critical for tissue accretion and linear growth [64]. In our sample, children already had BMI-for-age z-scores above the reference median. Within this context, shorter sleep duration was associated with higher BMI-related indicators and greater FFM. This dual pattern has also been described in other pediatric cohorts [65,66,67]. However, because absolute FFM scales with overall body size and total weight during early childhood, greater FFM may reflect differences in body size rather than a healthier composition profile and should be interpreted with caution [68]. These findings point to sleep as a potentially modifiable factor that should be considered within early obesity prevention strategies. 

Physical activity, in total and at moderate to vigorous intensity, displayed the strongest and most consistent positive associations with growth indicators and FFM, reinforcing the co-occurrence of physical activity with healthy somatic development parameters rather than direct reductions in fat accumulation [55,69]. However, given the cross-sectional design of this study, the direction of these associations cannot be established. Larger, heavier, or more biologically mature children may exhibit greater muscle strength and higher measured physical activity levels due, at least partly, to differences in body size and maturational status, rather than these characteristics necessarily resulting from greater physical activity. The stronger correlations with FFM than with fat mass or body fat percentage suggest that, in preschool years, activity may be more closely related to musculoskeletal development and bone mineralization, which is consistent with physiological expectations for this stage of life [70,71]. Evidence from international cohorts also shows that higher MVPA is related to greater lean body mass, while its associations with adiposity indicators are comparatively weaker [53,54,55]. In practical terms, this implies that interventions promoting MVPA at young ages should emphasize their broader potential associations with growth, functional capacity, and metabolic health beyond the sole objective of preventing fat gain. 

In this study, device-measured sedentary time was not significantly associated with adiposity or growth indicators, and parent-reported sitting time was likewise not significantly associated with height or weight. These findings do not indicate a directional discrepancy between the two measures. They are consistent with the distinction between total sedentary time and specific sitting domains. Device-measured sedentary time reflects a wide spectrum of low-energy activities (≤1.5 metabolic equivalents of task [METs]), many of which, such as reading, drawing, or structured seated learning, may not be detrimental to health and can even support cognitive development [32,72,73,74]. This heterogeneity likely weakens its associations with obesity, explaining why multiple systematic reviews have reported inconsistent findings in young children [58,75]. Conversely, screen-based behaviors carry unique risks because they are more consistently coupled with adverse exposures, including displacement of physical activity and sleep, passive overconsumption of calorie-dense foods, and exposure to marketing cues, all of which frequently co-occur with markers of energy imbalance and higher weight status [76,77,78,79,80]. Accurately parsing this sedentary-versus-active distinction in pediatric populations is therefore essential for interpreting lifestyle behaviors and obesity-related outcomes [81]. Furthermore, the observed positive association between parent-reported time spent outdoors and obesity represents an unexpected finding that requires cautious interpretation. This counterintuitive relationship may be explained by residual confounding, as parental reports of outdoor time capture overall exposure to outdoor environments rather than true active movement, potentially including prolonged sedentary behaviors (e.g., stroller transport, passive sitting, or outdoor screen exposure) [58]. Alternatively, reverse causality or social desirability bias may contribute to this pattern, as parents of children with higher weight status might more actively encourage or overreport outdoor activities [82].

The analysis of the WHO 24-hour movement guidelines offers a broader perspective on how daily behaviors relate to body composition [21,23,34,83]. In our study, full adherence defined through accelerometry showed a limited association, reaching significance only with FFM, while no associations were observed with fat mass or body fat percentage. This suggests that, in preschool children, adherence to the integrated 24-hour movement guidelines may be more closely linked to growth processes and lean tissue development than to adiposity outcomes [84]. This may be explained by the fact that FFM shows greater variability at this age and is more sensitive to behavioral influences, while fat mass changes more gradually and within a narrower range [34,54]. Moreover, during the preschool period, rapid linear growth and musculoskeletal development are biologically prioritized, which can dilute short-term associations with fat mass and make lean outcomes more responsive to daily behaviors [34,70,71]. Additionally, the cross-sectional design and small sample reduce the ability to detect subtle associations with adiposity [37,44]. Finally, prospective evidence suggests that the protective effects of integrated behaviors on fat accumulation may emerge later in childhood, whereas in early years they are primarily reflected in lean tissue accretion [65,66].

Accelerometer-derived and parent-reported measures address distinct constructs, which is a central methodological concern in movement behavior research [85]. Accelerometry provides valid estimates of duration, intensity, and temporal patterns, but lacks behavioral context such as the type of sedentary activity or bedtime practices [45,86]. In young children, movement patterns are characteristically sporadic, unstructured, and intermittent, consisting of short bursts of activity distributed throughout the day. High frequency accelerometry captures these spontaneous activity dynamics with high temporal resolution, minimizing misclassification of intensity and duration [44]. Parent questionnaires capture domain specific information on screen use and sleep hygiene, yet are susceptible to recall error, social desirability bias, and imprecise duration estimates [87]. Additionally, parents often struggle to accurately differentiate light activity from moderate-to-vigorous physical activity during unstandardized play, introducing measurement noise that can attenuate statistical associations with health outcomes [88]. Consequently, in this study, stronger and more consistent associations from accelerometer data support the value of objective measurement for evaluating relationships with somatic development and body composition in preschool children. The divergence between objective and subjective data favors a combined approach in which device-based monitoring delivers robust daily profiles and parental reports add contextual information for interpretation and intervention targeting [89,90]. Moving forward, future studies should adopt device-based assessment as the analytic core and incorporate well-designed questionnaires to improve epidemiological precision and ecological validity.

These preliminary findings may help generate hypotheses for early-life health promotion research in Ecuador and comparable settings. Family and preschool programs could be explored in future work regarding healthy sleep habits and opportunities for MVPA through active play. Evidence from structured physical activity interventions in pediatric populations also supports the potential relevance of this approach [91]. However, the present cross-sectional, largely unadjusted pilot analyses do not support causal inferences or definitive policy recommendations. Future research should use longitudinal and nationally representative designs, incorporate dietary as well as familial, cultural and school-related factors, and evaluate the feasibility of strategies aimed at improving adherence to 24-hour guidelines in diverse socioeconomic contexts.

This study has several notable strengths, including device-measured assessment of physical activity, sedentary time, and sleep; the balanced inclusion of urban and rural preschoolers; and its contribution as the first study of this kind in Ecuador conducted as part of the SUNRISE pilot study. Nevertheless, some limitations must be explicitly emphasized. First, the study relied on a non-probabilistic convenience sampling strategy restricted to specific educational centers in Quito and surrounding areas. Consequently, the sample lacks nationwide representativeness, and findings cannot be generalized to the broader Ecuadorian preschool population or other regions with distinct socioeconomic, geographic, and cultural dynamics. Second, the cross-sectional design precludes any causal inferences regarding the directionality of the observed associations. Consequently, these associations should not be definitively interpreted as protective or health-promoting developmental profiles until confirmed by prospective longitudinal studies capable of tracking bidirectional temporal pathways. Third, the small sample size inherently reduces statistical power, increasing the risk of type I and type II errors, which may artificially inflate or mask true associations, and precluded adequately powered sex-stratified analyses. Fourth, primary analyses relied on unadjusted bivariate correlations. Although complementary multivariable models with center-level cluster-robust standard errors were added for a limited set of primary associations (Supplementary Tables S4 and S5), residual confounding from unmeasured factors such as dietary intake, parental BMI, socioeconomic status, childcare attendance, family lifestyle and environmental characteristics cannot be excluded, and the pilot sample was underpowered for stable multivariable inference. Fifth, fat-free mass, fat mass and body fat percentage were estimated using a validated anthropometric prediction equation [42] rather than a criterion body-composition technique; therefore, correlations with size-related outcomes partly reflect shared anthropometric information and may overstate independent associations. Sixth, approximately 108 pairwise correlations were examined in Figure 1. We now report 95% CIs and apply Benjamini–Hochberg FDR control across the full unfiltered matrix (Supplementary Table S7): 44 associations were nominally significant and 30 remained at q < 0.05. Associations with |ρ| < 0.29 at n = 93, including the sleep–height coefficient, should be interpreted as statistically fragile even where *p* < 0.05. The parent-reported outdoor time–obesity association in Figure 2 was based on only eight obesity cases and was not FDR-controlled. Additionally, parent-reported data are subject to recall and social desirability biases. Dietary information, an important determinant of adiposity, was not systematically collected. Furthermore, participants were included with at least one valid accelerometer day; while this maximized participant retention according to the SUNRISE protocol applied in the primary study, these relatively permissive wear-time criteria may have reduced the reliability of habitual movement behavior estimates, although sensitivity analyses restricted to ≥2 valid days yielded similar adjusted estimates. Methodological aspects of accelerometry (e.g., device placement, epoch length, and cut-off criteria for activity intensities) may also affect comparability with other studies. Taken together, these limitations underscore the need for cautious interpretation, while reinforcing the study’s value as a preliminary contribution that generates hypotheses and highlights avenues for future, larger-scale research in Ecuador and comparable settings. Moreover, the generally small-to-moderate effect sizes observed in this study suggest that the reported associations should be interpreted with caution regarding their clinical significance, despite their potential relevance for understanding population-level movement behavior patterns in early childhood.

## 5. Conclusions

This secondary analysis of the SUNRISE Pilot Study Ecuador provides preliminary, hypothesis-generating evidence of associations between 24-hour movement behaviors and body composition indicators among Ecuadorian preschool children. Exploratory bivariate associations were identified for specific movement behaviors, including sleep duration and MVPA; however, these associations attenuated in complementary multivariable models. Due to the cross-sectional design, the exploratory nature of the primary unadjusted analyses and the equation-based estimation of body composition, these findings should be interpreted with caution and cannot establish causal relationships. These findings highlight the need for further research investigating individual movement behaviors, adherence to 24-hour movement guidelines, and integrated 24-hour movement behavior patterns using longitudinal designs and appropriate analytical approaches. Future studies including larger and nationally representative samples are needed to better understand the prospective associations between movement behaviors and body composition outcomes in Ecuadorian children.

## Figures and Tables

**Table 1:** Descriptive data of the study participants.

Variable	N = 108
Age (years)	4.0 (3.5, 4.3); 3.9 ± 0.6
Sex	
Boys	46 (42.6%)
Girls	62 (57.4%)
Area of residence	
Urban	55 (50.9%)
Rural	53 (49.1%)
Total sleep time (min) ^a^	714.0 (658.0, 759.5); 706.8 ± 89.2
Missing	15
Sedentary time (min)	492.6 (452.7, 522.7); 489.8 ± 62.0
Missing	15
LPA time (min)	100.4 (90.3, 116.7); 102.4 ± 21.3
Missing	15
MPA time (min)	58.4 (44.1, 71.3)
Missing	15
VPA time (min)	13.4 (8.4, 22.6)
Missing	15
MVPA time (min)	73.0 (53.1, 93.8); 76.4 ± 30.5
Missing	15
TPA time (min)	181.4 (144.5, 200.9); 178.8 ± 48.5
Missing	15
Sitting time (min)	90.0 (60.0, 120.0)
Missing	1
Total sleep time (min) ^b^	660.0 (600.0, 690.0); 651.6 ± 59.3
Missing	1
Time spent sitting in a vehicle (min)	38.6 (24.3, 62.1)
Missing	1
Time spent outdoors (min)	137.1 (77.1, 231.4)
Missing	1
MVPA guideline	
No	29 (31.2%)
Yes	64 (68.8%)
Missing	15
TPA guideline	
No	46 (49.5%)
Yes	47 (50.5%)
Missing	15
ST guideline	
No	56 (52.3%)
Yes	51 (47.7%)
Missing	1
Restrained sitting guideline	
No	43 (40.2%)
Yes	64 (59.8%)
Missing	1
Sleep guideline ^a^	
No	33 (35.5%)
Yes	60 (64.5%)
Missing	15
Sleep guideline ^b^	
No	15 (14.0%)
Yes	92 (86.0%)
Missing	1
All 24-hour component-level criteria ^c^	
None	2 (2.2%)
One	14 (15.1%)
Two	19 (20.4%)
Three	26 (28.0%)
Four	21 (22.6%)
Five	11 (11.8%)
Missing	15
All 24-hour component-level criteria ^d^	
None	2 (2.2%)
One	8 (8.6%)
Two	17 (18.3%)
Three	29 (31.2%)
Four	26 (28.0%)
Five	11 (11.8%)
Missing	15
Height (cm)	98.9 (96.0, 102.1); 98.9 ± 5.1
Weight (kg)	16.0 (14.5, 17.6); 16.3 ± 2.5
Height-for-age (z-score)	−0.8 (−1.5, −0.1); −0.8 ± 0.9
Weight-for-age (z-score)	0.0 (−0.7, 0.5); 0.0 ± 0.9
BMI-for-age (z-score) ^e^	0.7 (0.2, 1.3); 0.8 ± 0.9
BMI status	
Normal weight	66 (61.1%)
Overweight	34 (31.5%)
Obesity	8 (7.4%)
Excess weight status	
No excess weight	66 (61.1%)
Excess weight	42 (38.9%)
Fat-free mass (kg)	13.5 (12.7, 14.6); 13.7 ± 1.6
Fat mass (kg)	2.6 (1.9, 3.1); 2.6 ± 1.1
Body fat (%)	15.7 (12.5, 18.4); 15.5 ± 4.6

Data expressed as median (interquartile range) and mean ± standard deviation for continuous variables, and number (percentages) for categorical variables. BMI, body mass index; LPA, low-intensity physical activity; MPA, moderate-intensity physical activity; VPA, vigorous-intensity physical activity; MVPA, moderate to vigorous physical activity; TPA, total physical activity; ST, screen time. ^a^ Measured with an accelerometer. ^b^ Measured with a parent questionnaire. ^c^ Moderate to vigorous physical activity, total physical activity and sleep guidelines measured with an accelerometer; screen time and restrained sitting guidelines measured with a parent questionnaire. ^d^ Moderate to vigorous physical activity and total physical activity guidelines measured with an accelerometer; screen time, restrained sitting and sleep guidelines measured with a parent questionnaire. ^e^ According to the World Health Organization criteria. Fat-free mass, fat mass and body fat percentage were estimated using the Hudda et al. [42] predictive equation.

## Data Availability

The original contributions presented in this study are included in the article/Supplementary Materials. Further inquiries can be directed to the corresponding author.

## Supplementary Materials

The following supporting information can be downloaded at: https://www.mdpi.com/article/10.3390/children13091129/s1, Supplementary Material S1: STROBE Statement; Supplementary Material S2: Parent/Primary Caregiver Questionnaire; Supplementary Material S3: Missing data summary for variables used in the sensitivity analyses; Supplementary Material S4: Primary multivariable linear models for selected associations (cluster-robust standard errors by educational center); Supplementary Material S5: Sensitivity of adjusted primary models restricting to ≥2 valid accelerometer days; Supplementary Material S6: Spearman correlations for selected associations overall and by sex and area of residence (descriptive sensitivity analysis; unadjusted); Supplementary Material S7: Complete unfiltered Spearman correlation matrix for Figure 1 (12 movement x 9 anthropometric/body-composition indicators; pairwise complete cases).

## Abbreviations

The following abbreviations are used in this manuscript:

WHO	World Health Organization
SUNRISE	International Study of Movement Behaviors in the Early Years
UOW	University of Wollongong
REDCap	Research Electronic Data Capture
CI	Confidence interval
IQR	Interquartile range
SD	Standard deviation
r	Pearson correlation coefficient
ρ	Spearman’s rho
r_pb_	Point-biserial correlations
MET	Metabolic Equivalent of Task
LPA	Low intensity physical activity
MPA	Moderate-intensity physical activity
VPA	Vigorous-intensity physical activity
MVPA	Moderate to vigorous physical activity
TPA	Total physical activity
FM	Fat mass
FFM	Fat-free mass
CPM	Counts per minute
BMI	Body mass index
ST	Screen time
FDR	False discovery rate
NCD	Noncommunicable Diseases
TEM	Technical error of measurement
ICC	Intraclass correlation coefficient
STROBE	Strengthening the Reporting of Observational Studies in Epidemiology
