# Supplementary material for: Associations Between 24-Hour Movement Behaviors and Anthropometric and Body Composition Indicators in Ecuadorian Preschool Children: SUNRISE Pilot Study
Source: Children (Basel). 2026 Aug 24;13(9):1129. doi: 10.3390/children13091129 (PMC13605536; doi:10.3390/children13091129)

Supplementary Materials

Associations between 24-hour movement behaviors and anthropometric and body composition indicators in Ecuadorian preschool children: SUNRISE Pilot study Phase 3

Contents

1. S2. Parent/Primary Caregiver Questionnaire
2. S1. STROBE Statement
3. S3. Missing data summary
4. S6. Subgroup sensitivity correlations
5. S4. Primary multivariable models (cluster-robust SE by centre)
6. S5. Wear-time sensitivity (>=2 valid days)
7. S7. Spearman correlation matrix
8. Figures S1–S3

S1. STROBE Statement

Supplementary Material S1. STROBE Statement

	Item No	Recommendation	Reported on page no.
**Title and abstract**	1	(*a*) Indicate the study's design with a commonly used term in the title or the abstract	1
		(*b*) Provide in the abstract an informative and balanced summary of what was done and what was found	3
Introduction			
Background/rationale	2	Explain the scientific background and rationale for the investigation being reported	4-5
Objectives	3	State specific objectives, including any prespecified hypotheses	5
Methods			
Study design	4	Present key elements of study design early in the paper	6
Setting	5	Describe the setting, locations, and relevant dates, including periods of recruitment, exposure, follow-up, and data collection	6-7
Participants	6	(*a*) Give the eligibility criteria, and the sources and methods of selection of participants	6-7
Variables	7	Clearly define all outcomes, exposures, predictors, potential confounders, and effect modifiers. Give diagnostic criteria, if applicable	7-10
Data sources/ measurement	8*	For each variable of interest, give sources of data and details of methods of assessment (measurement). Describe comparability of assessment methods if there is more than one group	*7-10*
Bias	9	Describe any efforts to address potential sources of bias	10, 16
Study size	10	Explain how the study size was arrived at	6
Quantitative variables	11	Explain how quantitative variables were handled in the analyses. If applicable, describe which groupings were chosen and why	10
Statistical methods	12	(*a*) Describe all statistical methods, including those used to control for confounding	10-11
		(*b*) Describe any methods used to examine subgroups and interactions	Not applicable
		(*c*) Explain how missing data were addressed	11
		(*d*) If applicable, describe analytical methods taking account of sampling strategy	Not applicable
		(*e*) Describe any sensitivity analyses	Not applicable

Results			
Participants	13*	(a) Report numbers of individuals at each stage of study—eg numbers potentially eligible, examined for eligibility, confirmed eligible, included in the study, completing follow-up, and analysed	11-12
		(b) Give reasons for non-participation at each stage	6-7
		(c) Consider use of a flow diagram	Not applicable
Descriptive data	14*	(a) Give characteristics of study participants (eg demographic, clinical, social) and information on exposures and potential confounders	11-12, Table 1
		(b) Indicate number of participants with missing data for each variable of interest	11, Table 1
Outcome data	15*	Report numbers of outcome events or summary measures	11-13, Figures 1-3
Main results	16	(*a*) Give unadjusted estimates and, if applicable, confounder-adjusted estimates and their precision (eg, 95% confidence interval). Make clear which confounders were adjusted for and why they were included	12-13
		(*b*) Report category boundaries when continuous variables were categorized	7-10
		(*c*) If relevant, consider translating estimates of relative risk into absolute risk for a meaningful time period	Not applicable
Other analyses	17	Report other analyses done—eg analyses of subgroups and interactions, and sensitivity analyses	12-13
Discussion			
Key results	18	Summarise key results with reference to study objectives	13-14
Limitations	19	Discuss limitations of the study, taking into account sources of potential bias or imprecision. Discuss both direction and magnitude of any potential bias	16
Interpretation	20	Give a cautious overall interpretation of results considering objectives, limitations, multiplicity of analyses, results from similar studies, and other relevant evidence	13-16
Generalisability	21	Discuss the generalisability (external validity) of the study results	16-17
Other information			
Funding	22	Give the source of funding and the role of the funders for the present study and, if applicable, for the original study on which the present article is based	17

S2. Parent/Primary Caregiver Questionnaire

Supplementary Material S2. Parent/Primary Caregiver Questionnaire

International Study
of 24-hour movement behaviours in the early years

SUNRISE STUDY: INTERNATIONAL STUDY OF MOVEMENT-RELATED BEHAVIORS IN EARLY CHILDHOOD

PARENT/CAREGIVER QUESTIONNAIRE

This questionnaire must be administered/completed by the PRIMARY caregiver of the child living with him/her. At the time of the interview, the child must be 3 or 4 years old.

**CHILD AND PRIMARY CAREGIVER BACKGROUND MODULE**			
**1.**	What is the child's date of birth? (dd/mm/yyyy)	**2.**	If the date of birth is unknown. How old is the child? (number of years completed).
	_________/__________/__________		……………………………………...................................
**3.**	What is the relationship between the primary caregiver and the child participating in the study?		
	o Mother<br>o Father<br>o Grandmother<br>o Grandfather<br>o Legal Guardian<br>o Other (please specify):		
**4.**	What is the sex of the child?	**5.**	What is the gender of the primary caregiver?
	o Boy<br>o Girl		o Male<br>o Female<br>o Other<br>o Not Specified
**6.**	What is the primary caregiver's date of birth? (dd/mm/yyyy)	**7.**	If the primary caregiver does not know the date of birth, how old is he/she (in years)?
	_________/__________/__________		……………………………………............................
**8.**	What is the highest last level of instruction completed by a member of your household?		

	o None / Literacy Centre<br>o Early education<br>o Basic General Education<br>o Secondary Education / Baccalaureate<br>o Higher Education (Third Level / Fourth Level)<br>o Don't know/Don't answer
**9.**	How do you identify yourself according to your culture and customs? / What ethnic group do you feel identified with?
	o Afro-Ecuadorian<br>o Indigenous<br>o Mulatto<br>o Black<br>o White<br>o Mestizo<br>o Montuvio<br>o Other

**PHYSICAL ACTIVITY, SCREEN TIME, SEDENTARY BEHAVIOR AND SLEEP MODULE**		
The following questions pertain to the child's physical behaviors. Please indicate the number of hours and minutes per day for each. For example: 1 hour and 30 minutes.		
**10.**	In the **past week**, was there a day when the child was immobile for more than an hour continuously, either in a car, car seat, on the back, or on a motorcycle?	
	o Yes<br>o No	
**11.**	During a **typical day of the week**, how much time does the child spend as a passenger in a motor vehicle (e.g., a car, bus, motorcycle)?	.............h ....min
**12.**	During a **typical weekend** day, how much time does the child spend as a passenger in a motor vehicle (e.g., car, bus, motorcycle)?	.............h ....min
**13.**	On a typical day **of the week**, how much time does the child spend outdoors?	.............h ....min
**14.**	On a typical **weekend day**, how much time does the child spend outdoors?	.............h ....min
**15a.**	In the last three days, has the child left the house to play (alone, with an adult, or with an older child)?	
	o Yes<br>o No	

**15b.**	**If so**, where did the child go? **(Check as many as you feel are appropriate)**<br><br>o On the property (front garden, patio)<br>o To a friend or family member's house<br>o To a park, square or playground<br>o Swimming in a pool, river or beach<br>o To the street<br>o To the countryside, forest or natural environment<br>o Any other places not mentioned? (please specify):<br>………	
**15c.**	In the last three days, the child <u>has not gone or been allowed to go outside to play because</u> of (check all you consider):	
	o Heat<br>o Noise or crowd<br>o Cold<br>o Garbage or waste<br>o Rain<br>o Social unrest (e.g., protests)<br>o Air pollution (dirt, smoke, bad smell)<br>o Hazards such as assault, kidnapping, or crime<br>o Hazards due to vehicle traffic<br>o Water pollution<br>o Other not mentioned (please specify):<br>………<br>o Not important	
**16.**	**In a 24-hour period over the past week**, how much time did the child spend using any electronic display devices, such as a smartphone, tablet, video games, watching TV or movies, or videos on the Internet while sitting or lying down? Record this as accurately as possible to the nearest minute.	............h<br><br>.............min
**17.**	How often do you use screen devices to support your child's development or education (e.g., educational videos, apps, interactive games)?	
	o Never<br>o Less than once a week<br>o Once a week<br>o Most days<br>o Every day<br>o I don't know	
**18.**	How often do you use an electronic screen device to calm the child when he or she is upset?	

	o Never<br>o Less than once a week<br>o Once a week<br>o Most days<br>o Every day<br>o I don't know
**19.**	How often do you use an electronic display device to keep your child occupied while you do your errands?
	o Never<br>o Less than once a week<br>o Once a week<br>o Most days<br>o Every day<br>o I don't know
**20.**	How often do you use a smartphone to make calls, send text messages, check email, or watch a video **<u>during mealtimes with your child</u>**?
	o Never<br>o Less than once a week<br>o Once a week<br>o Most days<br>o Every day<br>o I don't know
**21.**	How often do you use a smartphone to make calls, send text messages, check email, or watch a video **<u>during playtime with the child</u>**?
	o Never<br>o Less than once a week<br>o Once a week<br>o Most days<br>o Every day<br>o I don't know
**22.**	How often do you use a smartphone to make calls, send text messages, check email, watch a video during **<u>travel/commute time with the child</u>**?
	o Never<br>o Less than once a week<br>o Once a week<br>o Most days<br>o Every day<br>o I don't know
**23.**	How often do you use a smartphone to make calls, send text messages, check email, watch a video **<u>while walking with the child</u>**?
	o Never<br>o Less than once a week<br>o Once a week<br>o Most days<br>o Every day<br>o I don't know

<table>
<tr><td>24.</td><td colspan="3">How often do you use a smartphone to make calls, send text messages, check email, watch a video <u>during your child's bedtime routine</u>?</td></tr>
<tr><td></td><td colspan="3">o Never
o Less than once a week
o Once a week
o Most days
o Every day
o I don't know</td></tr>
<tr><td>25a.</td><td colspan="3">Does the child use electronic display devices (e.g., television, video games, computer, tablet, or smartphone) daily <u>in the 2 hours before bedtime</u>?</td></tr>
<tr><td></td><td colspan="3">o Yes
o No
o I do not know</td></tr>
<tr><td>25b.</td><td colspan="3">If so, how close to bedtime does the child typically use these devices?</td></tr>
<tr><td></td><td colspan="3">o Less than 30 minutes before bedtime
o Between 30 minutes to 1 hour before bedtime
o 1-2 hours before bedtime</td></tr>
<tr><td>26.</td><td colspan="3">Does the child have electronic display devices in the room where he/she sleeps (e.g., TV, video games, computer, tablet, or smartphone)?</td></tr>
<tr><td></td><td colspan="3">o Yes
o No</td></tr>
<tr><td>27.</td><td colspan="2">How many hours does the child sleep in a typical 24-hour day (including naps)?</td><td>............h ....min</td></tr>
<tr><td>28.</td><td>Does the child nap?</td><td>28a.</td><td>If so, what time does the child nap?</td></tr>
<tr><td></td><td>o Yes
o No</td><td colspan="2">Starting time (24h format): ....
End time (24h format):....................</td></tr>
<tr><td>29a.</td><td>Does the child have a fixed bedtime?</td><td>29b.</td><td>Does the child have a fixed wake-up time?</td></tr>
<tr><td></td><td>o Yes, bedtime does not vary beyond 30 minutes each day.
o No, bedtime varies beyond 30 minutes each day.</td><td></td><td>o Yes, the time to wake up does not vary beyond 30 minutes each day.
o No, the time of waking up varies beyond 30 minutes each day.</td></tr>
<tr><td>30a.</td><td>What time does the child go to bed at night?</td><td>30b.</td><td>What time does the child get up in the morning?</td></tr>
</table>

	Bedtime (24h format):......................	Wake Up Time (24h Format):...............
**31.**	On a scale of 1 to 7, where the higher number indicates better sleep quality, how would you rate the child's sleep quality?<br>"1" would indicate a high difficulty in accommodating, wakes up many times during the night for prolonged periods and is very restless (tosses and turns, uncovers), while "7" would indicate that he settles in and falls asleep in a few minutes, sleeps through the night and has a very deep sleep.	
	o 1<br>o 2<br>o 3<br>o 4<br>o 5<br>o 6<br>o 7<br>o Don't know	
**32a.**	In the last three days, has the child slept enough?	
	o Yes<br>o No	
**32b.**	**If not.** Why was it? (check all you consider)	
	o Outside noise (vehicular traffic, trains, other street noises)<br>o Interior noise<br>o Very hot<br>o Very cold<br>o Lots of light coming into the room<br>o Other (please specify)..	
**33a.**	Does the child sleep alone in his/her own room?	
	o Yes<br>o No	
**33b.**	If the answer is **No**, how many other people sleep in the same room as the child?	
	………………… Children<br>…………………. Adults (18 years and older)	

**33c.**	If the answer is **No**, how many other people sleep in the same bed as the child?
	o No<br>..................... Children<br>…………………. Adults (18 years and older)
**34.**	How often does the child get into a bedtime routine (e.g., potty time, saying goodnight, telling stories, etc.)?
	o Never<br>o Less than once a week<br>o Once a week<br>o Most days<br>o Every day<br>o I don't know

**FOOD SOURCE MODULE**	
**35a.**	Did you ever give the child money to buy food?
	o Yes<br>o No
**35b.**	If the answer is **Yes**, how many days a week? _______ days
**36a.**	Does the child bring food to the school?
	o Yes<br>o No
**36b.**	If the answer is **Yes**, what food(s) do they usually eat and how many days a week?

	**Once a week**	**Twice a week**	**Three times a week**	**Four times a week**	**Five or more times a week**	**No**
**Breakfast**						
**Lunch**						
**Dinner**						

	**Collations**						

**37a.**	Does the child have any special dietary requirements/restrictions (e.g., dairy-free, gluten-free foods)?
	o Yes<br>o No
**37b.**	If the answer is **Yes**, please specify ______________________________

**FOOD DIVERSITY MODULE**	
**38.**	Did the child eat any of the following types of food **<u>yesterday, during the day, or at night</u>**?

	Yes	No	I don't know
**Grains, roots, and tubers (e.g., bread, cereals, pasta, pasta, potatoes, rice)**			
**Legumes and nuts (e.g., beans, beans, lentils, nuts, or seeds)**			
**Dairy/dairy products (e.g., cheese, ice cream, milk, yogurt, etc. Do not include butter or sour cream)**			
**Fresh foods (e.g., meat, fish, poultry, and liver/organ meats)**			
**Eggs**			
**Fruits and vegetables rich in vitamin A (e.g., leafy greens, yellow and orange vegetables inside, and orange fruits other than citrus)**			
**Other fruits and vegetables (e.g., apple, banana, orange, pear)**			

**EATING BEHAVIOR MODULE AT HOME**	
**39.**	How often is the TV or an electronic screen device turned on during lunch or snacks?
	o Never

	o Rarely<br>o Once a Week<br>o Most Days of the Week<br>o Every day<br>o I don't know
**40.**	How often do you sit with the child during meals?
	o Never<br>o Rarely<br>o Once a Week<br>o Most Days of the Week<br>o Every day<br>o I don't know
**41.**	How often does the whole family sit down during a main meal?
	o Never<br>o Rarely<br>o Once a Week<br>o Most Days of the Week<br>o Every day<br>o I don't know
**42.**	How often does the child eat or drink foods and drinks that are different from yours during meals and snacks?
	o Never<br>o Rarely<br>o Once a Week<br>o Most Days of the Week<br>o Every day<br>o I don't know
**43.**	How often does the child eat snacks such as chips, cookies, cakes, candy, chocolate, and candy between meals?
	o Never<br>o Rarely<br>o Once a Week<br>o Most Days of the Week<br>o Every day<br>o I don't know

**44.**	How often does the child consume sugary drinks (e.g., soda, flavored milk, flavored waters, fruit juices, beverages, etc.)?
	o Never<br>o Rarely<br>o Once a Week<br>o Most Days of the Week<br>o Every day<br>o I don't know

**FOOD INSECURITY MODULE**	
**45a.**	Have you ever run out of money to buy groceries in your home?
	o Yes<br>o No
**45b.**	Have you ever reduced the size of meals or skipped them because there isn't enough money?
	o Yes<br>o No
**45c.**	Do you go to bed hungry because there isn't enough money to buy food?
	o Yes<br>o No
**46.**	Feedback:<br>____<br>____<br>____<br>____<br>____________

**INDOOR AIR POLLUTION MODULE**	
**48.**	Is any food or drink consumed by household members cooked or prepared in the home using a stove, fire, or other cooking device?
	o Yes<br>o No

**49.**	How many stoves (including open fires) are used for these activities?
	o 1<br>o 2<br>o 3<br>o 4<br>o 5<br>o 6<br>o 7<br>o 8<br>o 9<br>o 10<br>o 11<br>o 12<br>o 13<br>o 14<br>o 15<br>o 16<br>o 17<br>o 18<br>o 19<br>o 20
**50.**	What type of cooking or method does this household primarily use to prepare food, boil water, or make hot beverages like tea or coffee?
	o 1. Solar cooker (which works with the heat of the sun, not with electric panels)<br>o 2. Electric cooker (resistance or induction)<br>o 3. Stove connected to natural gas (home network)<br>o 4. Biogas stove or stove (gas produced from organic waste)<br>o 5. Liquefied petroleum gas stove (gas in cylinder or cylinder)<br>o 6. Manufactured solid fuel stove (briquettes, pellets)<br>o 7. Traditional solid fuel stove (firewood, coal, agricultural residues)<br>o 8. Liquid fuel stove (kerosene, alcohol, or other flammable liquid)<br>o 9. Portable brazier (metal structure for direct fire cooking)<br>o 10. Three-stone hearth/open fire<br>o 11. Other (please specify)
**50a.**	Others, please specify:
**51. - 52.**	What is the main source of energy or fuel that this household uses for cooking, preparing food, making tea/coffee, or boiling water? **(Check as many as you feel are appropriate)**
	o 1. Alcohol/ethanol<br>o 2. Gasoline/diesel (not in generator)<br>o 3. Kerosene/paraffin<br>o 4. Unprocessed coal/lignite<br>o 5. Briquettes/coal pellets/lignite<br>o 6. Unprocessed charcoal

	o 7. Charcoal briquettes/pellets<br>o 8. Wood<br>o 9. Agricultural or crop residues/grass/straw/shrubs/corn cobs<br>o 10. Animal waste/manure<br>o 11. Processed biomass pellets/briquettes<br>o 12. Wood chips<br>o 13. Garbage/plastic<br>o 14. Sawdust<br>o 15. Other (please specify)
**51a. – 52a.**	Others, please specify:
**53.**	Do you usually cook in the house, in a separate building, or outdoors?
	o 1. In the main house: no separate room<br>o 2. In the main house: with separate room<br>o 3. Outside the main house: in a separate room<br>o 4. Outside the main outdoor house<br>o 5. On a terrace or covered porch<br>o 6. Other (please specify)
**53a.**	Others, please specify:
**54. – 62.**	Does the stove have a fireplace or extractor hood?
	o 1. Yes<br>o 0. No<br>o 2. I don't know
**55a. – 63.**	Are there other ways or methods used to cook in your community/village, in addition to those already mentioned?
	o 1. Yes, please specify<br>o 0. No
**55b. – 63a.**	Yes, please specify:
**56.**	Does this household use any heating or fire devices to keep the home/room warm at any time of the year?
	o Yes<br>o No

**57.**	In the last 12 months, for how many months did you use a heating or fire appliance to keep the home/room warm?
	o 0<br>o 1<br>o 2<br>o 3<br>o 4<br>o 5<br>o 6<br>o 7<br>o 8<br>o 9<br>o 10<br>o 11<br>o 12
**58.**	How many heaters or heating appliances (including stoves and fireplaces) are used in or around the home to produce heat at any time of the year?
	o 1<br>o 2<br>o 3<br>o 4<br>o 5<br>o 6<br>o 7<br>o 8<br>o 9<br>o 10<br>o 11<br>o 12<br>o 13<br>o 14<br>o 15<br>o 16<br>o 17<br>o 18<br>o 19<br>o 20
**59.**	What kind of equipment or method does your home use to heat the home when it's cold?
	o 1. Central heating system<br>o 2. Portable electric heater (manufactured)<br>o 3. Traditional heater (e.g. brazier, wood, charcoal)<br>o 4. Gas or electric stove (manufactured)<br>o 5. Traditional wood stove (homemade or non-industrial)<br>o 6. Portable pan or griddle used for heating<br>o 7. Stove or open fire (e.g., three-stone stove)

	o 8. Heat pump (reversible air conditioning)<br>o 9. Other, please specify.
**59a.**	Others, please specify:
**60. - 61.**	What is the main source of energy or fuel that this home uses to heat the space with a heater, stove, or other similar appliance? **(Check as many as you feel are appropriate)**
	o 1. Electricity (including solar panels)<br>o 2. Natural gas (piped/piped)<br>o 3. Cooking gas (LPG, cylinder)<br>o 4. Biogas<br>o 5. Alcohol/ethanol<br>o 6. Gasoline/diesel (not in generator)<br>o 7. Kerosene/paraffin<br>o 8. Unprocessed coal/lignite<br>o 9. Briquettes/coal pellets/lignite<br>o 10. Unprocessed charcoal<br>o 11. Charcoal briquettes/pellets<br>o 12. Wood or firewood<br>o 13. Agricultural residues (grass, straw, cobs, shrubs)<br>o 14. Animal waste/manure<br>o 15. Processed biomass pellets/briquettes<br>o 16. Wood chips<br>o 17. Garbage/plastic<br>o 18. Sawdust<br>o 19. Others, please specify
**60a. - 61 B.C.**	Others, please specify:
**64.**	Does your home use any type of lighting?
	o Yes<br>o No
**65.**	How many different light sources (such as lamps, spotlights, or light bulbs) are there in total in this home or room?
	o 1<br>o 2<br>o 3<br>o 4<br>o 5<br>o 6<br>o 7

	o 8<br>o 9<br>o 10<br>o 11<br>o 12<br>o 13<br>o 14<br>o 15<br>o 16<br>o 17<br>o 18<br>o 19<br>o 20
**66.**	What is the main source of energy or light that this home uses for lighting? (Select a type for each light source in Question 65)
	o 1. Electricity (including solar panels)<br>o 2. Solar lantern<br>o 3. Portable rechargeable flashlight<br>o 4. Battery operated flashlight<br>o 5. Biogas lamp<br>o 6. LPG lamp<br>o 7. Gasoline lamp<br>o 8. Kerosene/paraffin lamp<br>o 9. Oil lamp<br>o 10. Candle<br>o 11. Open fire<br>o 12. Other, please specify
**66a.**	Others, please specify:
**67.**	Are there other lighting sources used in the community/village that are not mentioned above?
	o 1. Yes, please specify<br>o 0. No
**67a.**	Yes, please specify:
**47. - 68.**	Date the survey was completed:<br>DD/MM/YYYY: ______/________/____________

S3. Missing data

Supplementary Table S3. Missing data summary for variables used in the sensitivity analyses.

**Variable**	**Missing, n**	**Total, n**	**Available, n**	**Missing, %**
Age (years)	0	108	108	0.0
Weight (kg)	0	108	108	0.0
BMI-for-age (z-score)	0	108	108	0.0
Fat-free mass (kg)	0	108	108	0.0
Device-measured sleep (min)	15	108	93	13.9
Device-measured MVPA (min)	15	108	93	13.9

BMI, body mass index; MVPA, moderate- to vigorous- physical activity.

S4–S5. Adjusted models and wear-time sensitivity

Supplementary Table S4. Primary multivariable linear models for selected associations (cluster-robust standard errors by educational centre). Body composition (FFM, FM, body fat %) was estimated with the Hudda et al. (2019) equation using weight, height, age, sex and ethnicity; therefore FFM/FM models additionally adjust for height. Estimates are beta coefficients per 1-unit increase in the exposure (sleep in hours/day; MVPA in min/day). These analyses are complementary to the main exploratory correlation matrices and remain hypothesis-generating.

Model	Specification	n	<ce><b2>	95% CI	p	R<c2><b2>
M1	Sleep (h) -> BAZ | age, sex, sector	93	-0.110	-0.273 to 0.052	0.187	0.064
M2	Sleep (h) -> weight | age, sex, sector, height	93	-0.131	-0.341 to 0.080	0.228	0.705
M3	Sleep (h) -> FFM | age, sex, sector, height	93	-0.083	-0.207 to 0.042	0.196	0.802
M4	Sleep (h) -> FM | age, sex, sector, height	93	-0.048	-0.140 to 0.044	0.312	0.638
M5	MVPA (min/d) -> BAZ | age, sex, sector	93	0.007	0.000 to 0.014	0.050	0.084
M6	MVPA (min/d) -> weight | age, sex, sector, height	93	0.008	-0.004 to 0.020	0.208	0.707
M7	MVPA (min/d) -> FFM | age, sex, sector, height	93	0.005	-0.001 to 0.011	0.120	0.803
M8	MVPA (min/d) -> FM | age, sex, sector, height	93	0.003	-0.003 to 0.009	0.341	0.640

Unadjusted linear counterparts (cluster-robust SE by centre):

Model	Specification	n	<ce><b2>	95% CI	p	R<c2><b2>
U1	Sleep (h) -> BAZ | unadjusted	93	-0.127	-0.300 to 0.045	0.151	0.045
U2	Sleep (h) -> weight | unadjusted	93	-0.408	-0.690 to -0.127	0.006	0.059
U3	Sleep (h) -> FFM | unadjusted	93	-0.317	-0.484 to -0.149	<0.001	0.086
U4	Sleep (h) -> FM | unadjusted	93	-0.092	-0.228 to 0.045	0.192	0.015
U5	MVPA (min/d) -> BAZ | unadjusted	93	0.008	0.001 to 0.015	0.035	0.067
U6	MVPA (min/d) -> weight | unadjusted	93	0.027	0.013 to 0.041	<0.001	0.109
U7	MVPA (min/d) -> FFM | unadjusted	93	0.021	0.012 to 0.030	<0.001	0.158
U8	MVPA (min/d) -> FM | unadjusted	93	0.006	0.000 to 0.012	0.050	0.028

Supplementary Table S5. Sensitivity of adjusted primary models restricting to children with >=2 valid accelerometer days versus the SUNRISE protocol criterion (>=1 valid day).

Model	Specification	n	<ce><b2>	95% CI	p	R<c2><b2>	Sample
M1	Sleep (h) -> BAZ | age, sex, sector	89	-0.142	-0.345 to 0.061	0.174	0.088	>=2 valid days
M1	Sleep (h) -> BAZ | age, sex, sector	93	-0.110	-0.273 to 0.052	0.187	0.064	>=1 valid day (protocol)
M2	Sleep (h) -> weight | age, sex, sector, height	89	-0.175	-0.464 to 0.114	0.239	0.722	>=2 valid days
M2	Sleep (h) -> weight | age, sex, sector, height	93	-0.131	-0.341 to 0.080	0.228	0.705	>=1 valid day (protocol)
M3	Sleep (h) -> FFM | age, sex, sector, height	89	-0.098	-0.271 to 0.075	0.271	0.814	>=2 valid days
M3	Sleep (h) -> FFM | age, sex, sector, height	93	-0.083	-0.207 to 0.042	0.196	0.802	>=1 valid day (protocol)
M4	Sleep (h) -> FM | age, sex, sector, height	89	-0.077	-0.196 to 0.042	0.207	0.653	>=2 valid days
M4	Sleep (h) -> FM | age, sex, sector, height	93	-0.048	-0.140 to 0.044	0.312	0.638	>=1 valid day (protocol)
M5	MVPA (min/d) -> BAZ | age, sex, sector	89	0.006	-0.001 to 0.013	0.115	0.076	>=2 valid days
M5	MVPA (min/d) -> BAZ | age, sex, sector	93	0.007	0.000 to 0.014	0.050	0.084	>=1 valid day (protocol)
M6	MVPA (min/d) -> weight | age, sex, sector, height	89	0.005	-0.006 to 0.016	0.373	0.716	>=2 valid days
M6	MVPA (min/d) -> weight | age, sex, sector, height	93	0.008	-0.004 to 0.020	0.208	0.707	>=1 valid day (protocol)
M7	MVPA (min/d) -> FFM | age, sex, sector, height	89	0.003	-0.002 to 0.009	0.233	0.811	>=2 valid days
M7	MVPA (min/d) -> FFM | age, sex, sector, height	93	0.005	-0.001 to 0.011	0.120	0.803	>=1 valid day (protocol)

Model	Specification	n	<ce><b2>	95% CI	p	R<c2><b2>	Sample
M8	MVPA (min/d) -> FM ∣ age, sex, sector, height	89	0.002	-0.004 to 0.007	0.572	0.646	>=2 valid days
M8	MVPA (min/d) -> FM ∣ age, sex, sector, height	93	0.003	-0.003 to 0.009	0.341	0.640	>=1 valid day (protocol)

S6. Subgroup sensitivity tables

Supplementary Table S6. Spearman correlations for selected associations overall and by sex and area of residence (descriptive sensitivity analysis; unadjusted).

**Association**	**Subgroup**	**n**	**ρ**	**p-value**	**Direction**	**Direction consistent with overall**
Sleep vs weight	Overall	93	-0.33	0.001	Negative	Reference
Sleep vs weight	Sex: Male	43	-0.28	0.065	Negative	Yes
Sleep vs weight	Sex: Female	50	-0.25	0.084	Negative	Yes
Sleep vs weight	Area: Urban	48	-0.06	0.663	Negative	Yes
Sleep vs weight	Area: Rural	45	-0.59	<0.001	Negative	Yes
Sleep vs BMI z-score	Overall	93	-0.29	0.005	Negative	Reference
Sleep vs BMI z-score	Sex: Male	43	-0.5	0.001	Negative	Yes
Sleep vs BMI z-score	Sex: Female	50	-0.09	0.543	Negative	Yes
Sleep vs BMI z-score	Area: Urban	48	-0.12	0.398	Negative	Yes
Sleep vs BMI z-score	Area: Rural	45	-0.4	0.007	Negative	Yes
Sleep vs FFM	Overall	93	-0.37	<0.001	Negative	Reference
Sleep vs FFM	Sex: Male	43	-0.27	0.084	Negative	Yes
Sleep vs FFM	Sex: Female	50	-0.29	0.039	Negative	Yes
Sleep vs FFM	Area: Urban	48	-0.18	0.221	Negative	Yes
Sleep vs FFM	Area: Rural	45	-0.55	<0.001	Negative	Yes
MVPA vs FFM	Overall	93	0.44	<0.001	Positive	Reference
MVPA vs FFM	Sex: Male	43	0.27	0.081	Positive	Yes
MVPA vs FFM	Sex: Female	50	0.46	0.001	Positive	Yes
MVPA vs FFM	Area: Urban	48	0.44	0.002	Positive	Yes

Association	Subgroup	n	ϱ	p-value	Direction	Direction consistent with overall
MVPA vs FFM	Area: Rural	45	0.45	0.002	Positive	Yes
MVPA vs weight	Overall	93	0.4	<0.001	Positive	Reference
MVPA vs weight	Sex: Male	43	0.29	0.059	Positive	Yes
MVPA vs weight	Sex: Female	50	0.4	0.004	Positive	Yes
MVPA vs weight	Area: Urban	48	0.38	0.008	Positive	Yes
MVPA vs weight	Area: Rural	45	0.45	0.002	Positive	Yes

Note. For subgroup rows, "Direction consistent with overall" compares the sign of rho with the overall estimate: Yes = same direction; No = opposite direction; Not estimable = correlation could not be computed in that subgroup (n < 3 or missing data); Reference = overall row. Spearman's rho (ϱ).

S7. Spearman correlation matrix

Supplementary Table S7. Complete unfiltered Spearman correlation matrix for Figure 1 (12 movement x 9 anthropometric/body-composition indicators; pairwise complete cases). 95% CIs were obtained by Fisher's z transformation. q-values are Benjamini-Hochberg false-discovery-rate adjusted across the full family of 108 tests. Primary narrative reporting remains exploratory; BH-significant cells are a robustness overlay, not a replacement of the unadjusted heatmaps.

Movement	Outcome	n	rho	95% CI	p	q (BH)	BH q<0.05
Device-measured sleep (min)	Height (cm)	93	-0.20	-0.39 to -0.00	0.049	0.100	No
Sedentary time (min)	Height (cm)	93	0.15	-0.06 to 0.34	0.154	0.227	No
LPA (min)	Height (cm)	93	0.16	-0.05 to 0.35	0.134	0.207	No
MPA (min)	Height (cm)	93	0.32	0.12 to 0.49	0.002	0.012	Yes
VPA (min)	Height (cm)	93	0.27	0.07 to 0.45	0.009	0.029	Yes
MVPA (min)	Height (cm)	93	0.32	0.13 to 0.49	0.002	0.011	Yes
TPA (min)	Height (cm)	93	0.27	0.07 to 0.45	0.008	0.029	Yes
Parent-reported restrained time (min)	Height (cm)	107	-0.00	-0.19 to 0.19	0.995	0.995	No
Parent-reported sitting time (min)	Height (cm)	107	0.15	-0.04 to 0.33	0.124	0.199	No
Parent-reported sleep (min)	Height (cm)	107	-0.25	-0.42 to -0.06	0.010	0.032	Yes
Parent-reported vehicle time (min)	Height (cm)	0	NA	NA			No
Parent-reported outdoor time (min)	Height (cm)	0	NA	NA			No

Movement	Outcome	n	rho	95% CI	p	q (BH)	BH q<0.05
Device-measured sleep (min)	Weight (kg)	93	-0.33	-0.50 to -0.14	0.001	0.010	Yes
Sedentary time (min)	Weight (kg)	93	0.20	-0.00 to 0.39	0.053	0.105	No
LPA (min)	Weight (kg)	93	0.27	0.07 to 0.45	0.009	0.029	Yes
MPA (min)	Weight (kg)	93	0.41	0.23 to 0.57	<0.001	0.001	Yes
VPA (min)	Weight (kg)	93	0.33	0.13 to 0.50	0.001	0.010	Yes
MVPA (min)	Weight (kg)	93	0.40	0.22 to 0.56	<0.001	0.001	Yes
TPA (min)	Weight (kg)	93	0.38	0.19 to 0.54	<0.001	0.002	Yes
Parent-reported restrained time (min)	Weight (kg)	107	0.07	-0.12 to 0.25	0.485	0.560	No
Parent-reported sitting time (min)	Weight (kg)	107	0.12	-0.08 to 0.30	0.234	0.320	No
Parent-reported sleep (min)	Weight (kg)	107	-0.22	-0.39 to -0.03	0.022	0.055	No
Parent-reported vehicle time (min)	Weight (kg)	0	NA	NA			No
Parent-reported outdoor time (min)	Weight (kg)	0	NA	NA			No
Device-measured sleep (min)	BMI (kg/m2)	93	-0.28	-0.46 to -0.08	0.006	0.026	Yes
Sedentary time (min)	BMI (kg/m2)	93	0.13	-0.08 to 0.32	0.221	0.307	No
LPA (min)	BMI (kg/m2)	93	0.24	0.04 to 0.42	0.020	0.051	No
MPA (min)	BMI (kg/m2)	93	0.27	0.07 to 0.45	0.009	0.029	Yes
VPA (min)	BMI (kg/m2)	93	0.18	-0.02 to 0.37	0.084	0.145	No
MVPA (min)	BMI (kg/m2)	93	0.25	0.05 to 0.43	0.016	0.046	Yes
TPA (min)	BMI (kg/m2)	93	0.28	0.08 to 0.46	0.006	0.026	Yes
Parent-reported restrained time (min)	BMI (kg/m2)	107	0.09	-0.10 to 0.27	0.369	0.468	No
Parent-reported sitting time (min)	BMI (kg/m2)	107	0.02	-0.17 to 0.21	0.817	0.842	No
Parent-reported sleep (min)	BMI (kg/m2)	107	-0.07	-0.26 to 0.12	0.470	0.550	No
Parent-reported vehicle time (min)	BMI (kg/m2)	0	NA	NA			No
Parent-reported outdoor time (min)	BMI (kg/m2)	0	NA	NA			No
Device-measured sleep (min)	Weight-for-age z	93	-0.27	-0.45 to -0.07	0.008	0.029	Yes
Sedentary time (min)	Weight-for-age z	93	0.19	-0.01 to 0.38	0.068	0.125	No
LPA (min)	Weight-for-age z	93	0.22	0.02 to 0.41	0.032	0.071	No
MPA (min)	Weight-for-age z	93	0.34	0.15 to 0.51	<0.001	0.008	Yes
VPA (min)	Weight-for-age z	93	0.28	0.08 to 0.46	0.007	0.029	Yes

Movement	Outcome	n	rho	95% CI	p	q (BH)	BH q<0.05
MVPA (min)	Weight-for-age z	93	0.34	0.15 to 0.51	<0.001	0.008	Yes
TPA (min)	Weight-for-age z	93	0.31	0.11 to 0.48	0.002	0.015	Yes
Parent-reported restrained time (min)	Weight-for-age z	107	0.10	-0.09 to 0.28	0.307	0.406	No
Parent-reported sitting time (min)	Weight-for-age z	107	0.08	-0.11 to 0.27	0.407	0.489	No
Parent-reported sleep (min)	Weight-for-age z	107	-0.15	-0.33 to 0.05	0.136	0.207	No
Parent-reported vehicle time (min)	Weight-for-age z	0	NA	NA			No
Parent-reported outdoor time (min)	Weight-for-age z	0	NA	NA			No
Device-measured sleep (min)	Height-for-age z	93	-0.17	-0.36 to 0.04	0.110	0.184	No
Sedentary time (min)	Height-for-age z	93	0.14	-0.07 to 0.33	0.195	0.284	No
LPA (min)	Height-for-age z	93	0.09	-0.12 to 0.28	0.413	0.489	No
MPA (min)	Height-for-age z	93	0.24	0.04 to 0.42	0.019	0.051	No
VPA (min)	Height-for-age z	93	0.22	0.01 to 0.40	0.037	0.079	No
MVPA (min)	Height-for-age z	93	0.26	0.06 to 0.44	0.013	0.038	Yes
TPA (min)	Height-for-age z	93	0.18	-0.02 to 0.37	0.080	0.141	No
Parent-reported restrained time (min)	Height-for-age z	107	0.04	-0.15 to 0.23	0.672	0.720	No
Parent-reported sitting time (min)	Height-for-age z	107	0.08	-0.11 to 0.27	0.409	0.489	No
Parent-reported sleep (min)	Height-for-age z	107	-0.18	-0.36 to 0.01	0.059	0.112	No
Parent-reported vehicle time (min)	Height-for-age z	0	NA	NA			No
Parent-reported outdoor time (min)	Height-for-age z	0	NA	NA			No
Device-measured sleep (min)	BMI-for-age z	93	-0.29	-0.46 to -0.09	0.005	0.026	Yes
Sedentary time (min)	BMI-for-age z	93	0.13	-0.07 to 0.33	0.206	0.294	No
LPA (min)	BMI-for-age z	93	0.25	0.05 to 0.43	0.017	0.047	Yes
MPA (min)	BMI-for-age z	93	0.29	0.09 to 0.46	0.005	0.026	Yes
VPA (min)	BMI-for-age z	93	0.20	-0.01 to 0.39	0.057	0.112	No
MVPA (min)	BMI-for-age z	93	0.27	0.07 to 0.45	0.010	0.032	Yes
TPA (min)	BMI-for-age z	93	0.30	0.10 to 0.47	0.004	0.023	Yes
Parent-reported restrained time (min)	BMI-for-age z	107	0.10	-0.09 to 0.28	0.318	0.414	No
Parent-reported sitting time (min)	BMI-for-age z	107	0.02	-0.17 to 0.21	0.818	0.842	No
Parent-reported sleep (min)	BMI-for-age z	107	-0.08	-0.27 to 0.11	0.410	0.489	No

Movement	Outcome	n	rho	95% CI	p	q (BH)	BH q<0.05
Parent-reported vehicle time (min)	BMI-for-age z	0	NA	NA			No
Parent-reported outdoor time (min)	BMI-for-age z	0	NA	NA			No
Device-measured sleep (min)	Fat-free mass (kg)	93	-0.37	-0.53 to -0.18	<0.001	0.003	Yes
Sedentary time (min)	Fat-free mass (kg)	93	0.22	0.02 to 0.41	0.032	0.071	No
LPA (min)	Fat-free mass (kg)	93	0.29	0.09 to 0.46	0.005	0.026	Yes
MPA (min)	Fat-free mass (kg)	93	0.44	0.26 to 0.59	<0.001	<0.001	Yes
VPA (min)	Fat-free mass (kg)	93	0.37	0.18 to 0.53	<0.001	0.003	Yes
MVPA (min)	Fat-free mass (kg)	93	0.44	0.26 to 0.59	<0.001	<0.001	Yes
TPA (min)	Fat-free mass (kg)	93	0.41	0.22 to 0.56	<0.001	0.001	Yes
Parent-reported restrained time (min)	Fat-free mass (kg)	107	0.11	-0.08 to 0.29	0.265	0.357	No
Parent-reported sitting time (min)	Fat-free mass (kg)	107	0.04	-0.15 to 0.23	0.669	0.720	No
Parent-reported sleep (min)	Fat-free mass (kg)	107	-0.16	-0.34 to 0.03	0.095	0.161	No
Parent-reported vehicle time (min)	Fat-free mass (kg)	0	NA	NA			No
Parent-reported outdoor time (min)	Fat-free mass (kg)	0	NA	NA			No
Device-measured sleep (min)	Fat mass (kg)	93	-0.16	-0.35 to 0.05	0.133	0.207	No
Sedentary time (min)	Fat mass (kg)	93	0.13	-0.08 to 0.32	0.222	0.307	No
LPA (min)	Fat mass (kg)	93	0.15	-0.05 to 0.35	0.139	0.209	No
MPA (min)	Fat mass (kg)	93	0.25	0.04 to 0.43	0.018	0.048	Yes
VPA (min)	Fat mass (kg)	93	0.16	-0.04 to 0.35	0.124	0.199	No
MVPA (min)	Fat mass (kg)	93	0.23	0.02 to 0.41	0.030	0.069	No
TPA (min)	Fat mass (kg)	93	0.23	0.03 to 0.41	0.028	0.066	No
Parent-reported restrained time (min)	Fat mass (kg)	107	-0.01	-0.20 to 0.18	0.945	0.955	No
Parent-reported sitting time (min)	Fat mass (kg)	107	0.18	-0.01 to 0.36	0.064	0.121	No
Parent-reported sleep (min)	Fat mass (kg)	107	-0.22	-0.39 to -0.03	0.024	0.059	No
Parent-reported vehicle time (min)	Fat mass (kg)	0	NA	NA			No
Parent-reported outdoor time (min)	Fat mass (kg)	0	NA	NA			No
Device-measured sleep (min)	Body fat (%)	93	-0.04	-0.24 to 0.16	0.691	0.731	No
Sedentary time (min)	Body fat (%)	93	0.06	-0.15 to 0.26	0.575	0.639	No
LPA (min)	Body fat (%)	93	0.06	-0.14 to 0.26	0.564	0.634	No

Movement	Outcome	n	rho	95% CI	p	q (BH)	BH q<0.05
MPA (min)	Body fat (%)	93	0.09	-0.11 to 0.29	0.368	0.468	No
VPA (min)	Body fat (%)	93	0.02	-0.18 to 0.23	0.823	0.842	No
MVPA (min)	Body fat (%)	93	0.07	-0.13 to 0.27	0.495	0.564	No
TPA (min)	Body fat (%)	93	0.09	-0.11 to 0.29	0.377	0.472	No
Parent-reported restrained time (min)	Body fat (%)	107	-0.05	-0.24 to 0.14	0.600	0.659	No
Parent-reported sitting time (min)	Body fat (%)	107	0.17	-0.02 to 0.35	0.074	0.133	No
Parent-reported sleep (min)	Body fat (%)	107	-0.20	-0.37 to -0.01	0.044	0.092	No
Parent-reported vehicle time (min)	Body fat (%)	0	NA	NA			No
Parent-reported outdoor time (min)	Body fat (%)	0	NA	NA			No

Selected associations cited in the Results text (pairwise n; Fisher z 95% CI; BH q across 108 tests):

Association	n	rho	95% CI	p	q (BH)
Device-measured sleep (min) vs Height (cm)	93	-0.20	-0.39 to -0.00	0.049	0.100
Device-measured sleep (min) vs Weight (kg)	93	-0.33	-0.50 to -0.14	0.001	0.010
MVPA (min) vs Weight (kg)	93	0.40	0.22 to 0.56	<0.001	0.001
TPA (min) vs Weight (kg)	93	0.38	0.19 to 0.54	<0.001	0.002
Device-measured sleep (min) vs BMI (kg/m2)	93	-0.28	-0.46 to -0.08	0.006	0.026
Device-measured sleep (min) vs BMI-for-age z	93	-0.29	-0.46 to -0.09	0.005	0.026
Device-measured sleep (min) vs Fat-free mass (kg)	93	-0.37	-0.53 to -0.18	<0.001	0.003
Sedentary time (min) vs Fat-free mass (kg)	93	0.22	0.02 to 0.41	0.032	0.071
MVPA (min) vs Fat-free mass (kg)	93	0.44	0.26 to 0.59	<0.001	<0.001
TPA (min) vs Fat-free mass (kg)	93	0.41	0.22 to 0.56	<0.001	0.001

Figures S1–S3

Figure S1. Associations between 24-hour movement behaviors and body composition among Ecuadorian preschoolers. Colors: blue indicates a positive association and red a negative association. Color intensity reflects the magnitude of the correlation. Asterisks denote statistical significance based on unadjusted p-values: * p < 0.05, ** p < 0.01, *** p < 0.001; cells without asterisks were not significant. Abbreviations: BMI, body mass index; PA, physical activity. Measurement sources: a Measured with an accelerometer, b Measured with a parent questionnaire. P-values are uncorrected for multiple comparisons.

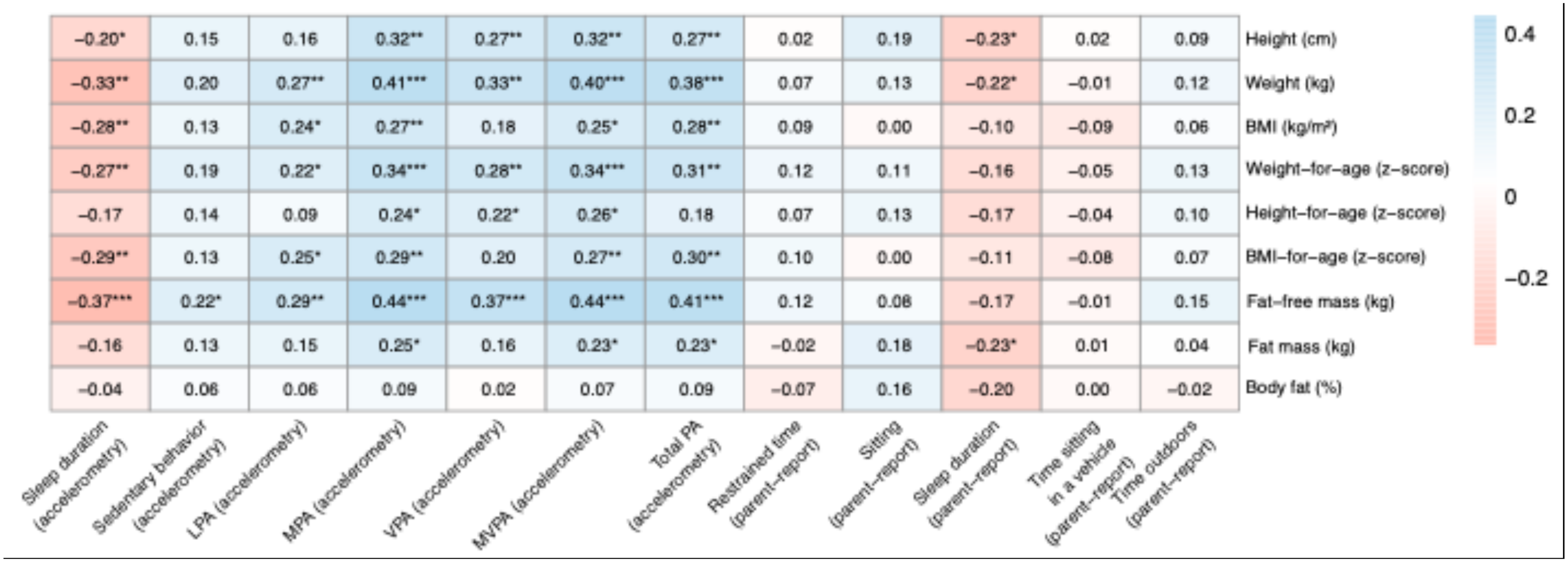

Figure S2. Associations between 24-hour movement behaviors and weight status among Ecuadorian preschoolers. Colors: blue indicates a positive association and red a negative association. Color intensity reflects the magnitude of the correlation. Asterisks denote statistical significance based on unadjusted p-values: * $p < 0.05$, ** $p < 0.01$, *** $p < 0.001$; cells without asterisks were not significant. Abbreviations: BMI, body mass index; PA, physical activity. Measurement sources: a Measured with an accelerometer, b Measured with a parent questionnaire. P-values are uncorrected for multiple comparisons.

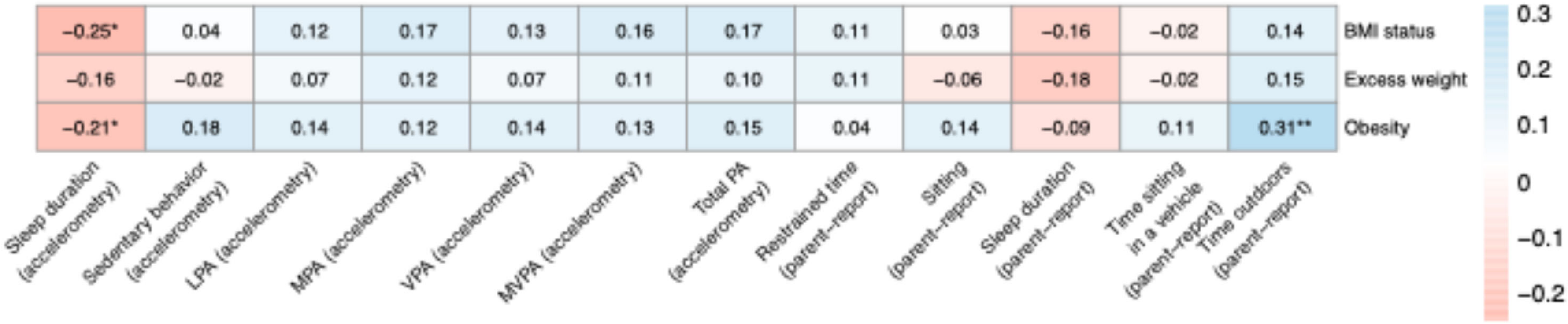

Figure S3. Compliance with the 24-hour movement guidelines and their relation to body composition among Ecuadorian preschoolers. Colors: blue denotes a positive association and red a negative association. Color intensity reflects the magnitude of the correlation. Asterisks indicate statistical significance based on unadjusted p-values: * $p < 0.05$, ** $p < 0.01$, *** $p < 0.001$; cells without asterisks were not significant. Abbreviations: BMI, body mass index; PA, physical activity. Measurement sources: a Measured with an accelerometer, b Measured with a parent questionnaire, c Moderate-to vigorous physical activity, total physical activity and sleep guidelines measured with an accelerometer; screen time and restrained sitting guidelines measured with a parent questionnaire, d Moderate-to vigorous physical activity and total physical activity guidelines measured with an accelerometer; screen time, restrained sitting and sleep guidelines measured with a parent questionnaire, e According to the World Health Organization criteria. P-values are uncorrected for multiple comparisons.

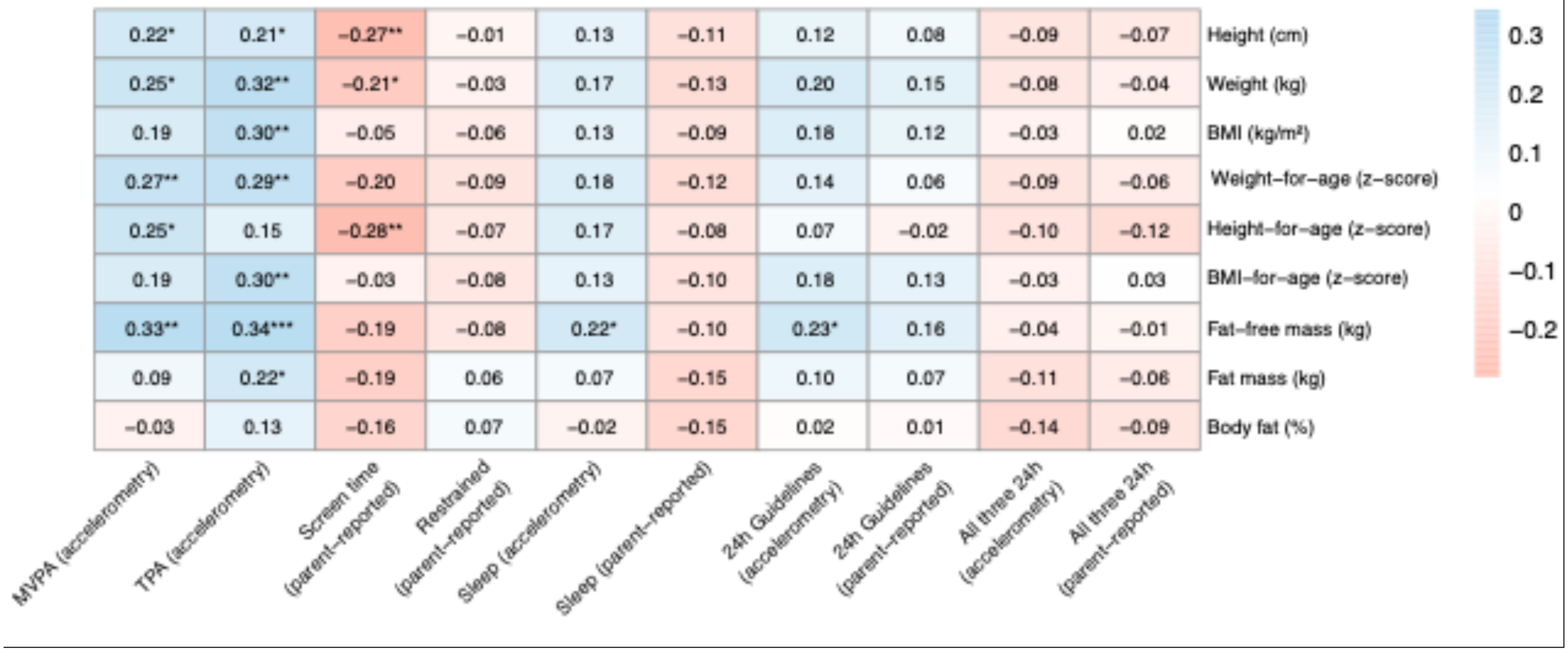

Supplement: Supplementary file 1 [file children-13-01129-s001.zip › children-4466925-supplementary.pdf]
